# Validation of quantitative [^18^F]NaF PET uptake parameters in bone diseases: a systematic review

**DOI:** 10.1007/s12149-024-01991-9

**Published:** 2024-12-27

**Authors:** Ruben D. de Ruiter, Jolien Zwama, Pieter G. H. M. Raijmakers, Maqsood Yaqub, George L. Burchell, Ronald Boellaard, Adriaan A. Lammertsma, Elisabeth M. W. Eekhoff

**Affiliations:** 1 Department of Endocrinology and Metabolism, Rare Bone Disease Center, Amsterdam University Medical Centers (UMC), Vrije Universiteit, Amsterdam Movement Sciences, Amsterdam, The Netherlands; 2 Department of Radiology and Nuclear Medicine, Amsterdam University Medical Centers (UMC), Vrije Universiteit, Amsterdam, The Netherlands; 3 University Library, Vrije Universiteit, Amsterdam, The Netherlands; 4 Nuclear Medicine and Molecular Imaging, University of Groningen, University Medical Center Groningen, Groningen, The Netherlands

**Keywords:** ^18^-Fluoride, PET, Bone diseases, [^18^F] NaF, Quantification, Sodiumfluoride

## Abstract

**Purpose:**

[^18^F]NaF PET has become an increasingly important tool in clinical practice toward understanding and evaluating diseases and conditions in which bone metabolism is disrupted. Full kinetic analysis using nonlinear regression (NLR) with a two-tissue compartment model to determine the net rate of influx (*K*_*i*_) of [^18^F]NaF is considered the gold standard for quantification of [^18^F]NaF uptake. However, dynamic scanning often is impractical in a clinical setting, leading to the development of simplified semi-quantitative parameters. This systematic review investigated which uptake parameters have been used to evaluate bone disorders and how they have been validated to measure disease activity.

**Methods:**

A literature search (in PubMed, Embase.com, and Clarivate Analytics/Web of Science Core Collection) was performed up to 28th November 2023, in collaboration with an information specialist. Each database was searched for relevant literature regarding the use of [^18^F]NAF PET/CT to measure disease activity in bone-related disorders. The main aim was to explore whether the reported semi-quantitative uptake values were validated against full kinetic analysis. A second aim was to investigate whether the chosen uptake parameter correlated with a disease-specific outcome or marker, validating its use as a clinical outcome or disease marker.

**Results:**

The initial search included 1636 articles leading to 92 studies spanning 29 different bone-related conditions in which [^18^F]NaF PET was used to quantify [^18^F]NaF uptake. In 12 bone-related disorders, kinetic analysis was performed and compared with simplified uptake parameters. SUV_mean_ (standardized uptake value) and SUV_max_ were used most frequently, though normalization of these values varied greatly between studies. In some disorders, various studies were performed evaluating [^18^F]NaF uptake as a marker of bone metabolism, but unfortunately, not all studies used this same approach, making it difficult to compare results between those studies.

**Conclusion:**

When using [^18^F]NaF PET to evaluate disease activity or treatment response in various bone-related disorders, it is essential to detail scanning protocols and analytical procedures. The most accurate outcome parameter can only be obtained through kinetic analysis and is better suited for research. Simplified uptake parameters are better suited for routine clinical practice and repeated measurements.

**Supplementary Information:**

The online version contains supplementary material available at 10.1007/s12149-024-01991-9.

## Introduction

[^18^F]NaF PET scanning has become an increasingly important tool in understanding and evaluating diseases and conditions in which bone metabolism is disrupted. In 1962, Blau et al. first demonstrated that there was increased uptake of ^18^F in areas of new bone formation, as compared with normal bone [1]. Since this first report, [^18^F]NaF PET has been established as an imaging modality for understanding and measuring treatment response in various metabolic bone disorders [2].

Bone formation typically occurs in two separate manners, i.e., as endochondral or as intramembranous ossification. In endochondral ossification, mesenchymal stem cells are stimulated to differentiate into chondrocytes, thereby creating a cartilage scaffold. After the cartilage scaffold has been established, mesenchymal stem cells differentiate into osteoblasts and start to produce an extracellular bone matrix, which slowly but steadily replaces the cartilage scaffold [3]. The second is through intramembranous ossification, in which mesenchymal stem cells differentiate directly into osteoblasts, which similarly create an extracellular bone matrix, but without first creating a cartilage scaffold. In the extracellular bone matrix, hydroxyapatite crystals are formed. After injection, [^18^F]NaF is distributed throughout the body and eventually binds to the crystallized surface, replacing the hydroxyl ions in hydroxyapatite to form fluorapatite [4, 5]. The tracer eventually accumulates in all sites of accessible bone, including sites of bone formation and bone degradation. The rate of accumulation depends on tracer availability, regional blood flow, and bone turnover [6].

[^18^F]NaF PET cannot only visualize, but also quantify areas of increased bone turnover. The pharmacokinetics of ^18^F-fluoride can best be described by a two-tissue compartment model for irreversible binding. This model was first described by Hawkins et al. in 1992 and since then it has been recognized as the gold standard for quantifying [^18^F]NaF uptake [6, 7]. This model considers plasma delivery of ^18^F-fluoride, its extraction fraction and, finally, its binding to bone matrix. Upon defining an area of interest, the net rate of transfer from plasma to bone binding (*K*_*i*_) can be estimated with a 60-min full dynamic scanning protocol, including arterial sampling, through nonlinear regression analysis (NLR). Even though this is the most accurate method for quantifying skeletal ^18^F-fluoride uptake, the procedure is less feasible in clinical practice due to the limited axial field of view covered by a dynamic scan, the complexity of data acquisition, and the burden it places on patients.

**Fig. 1:**
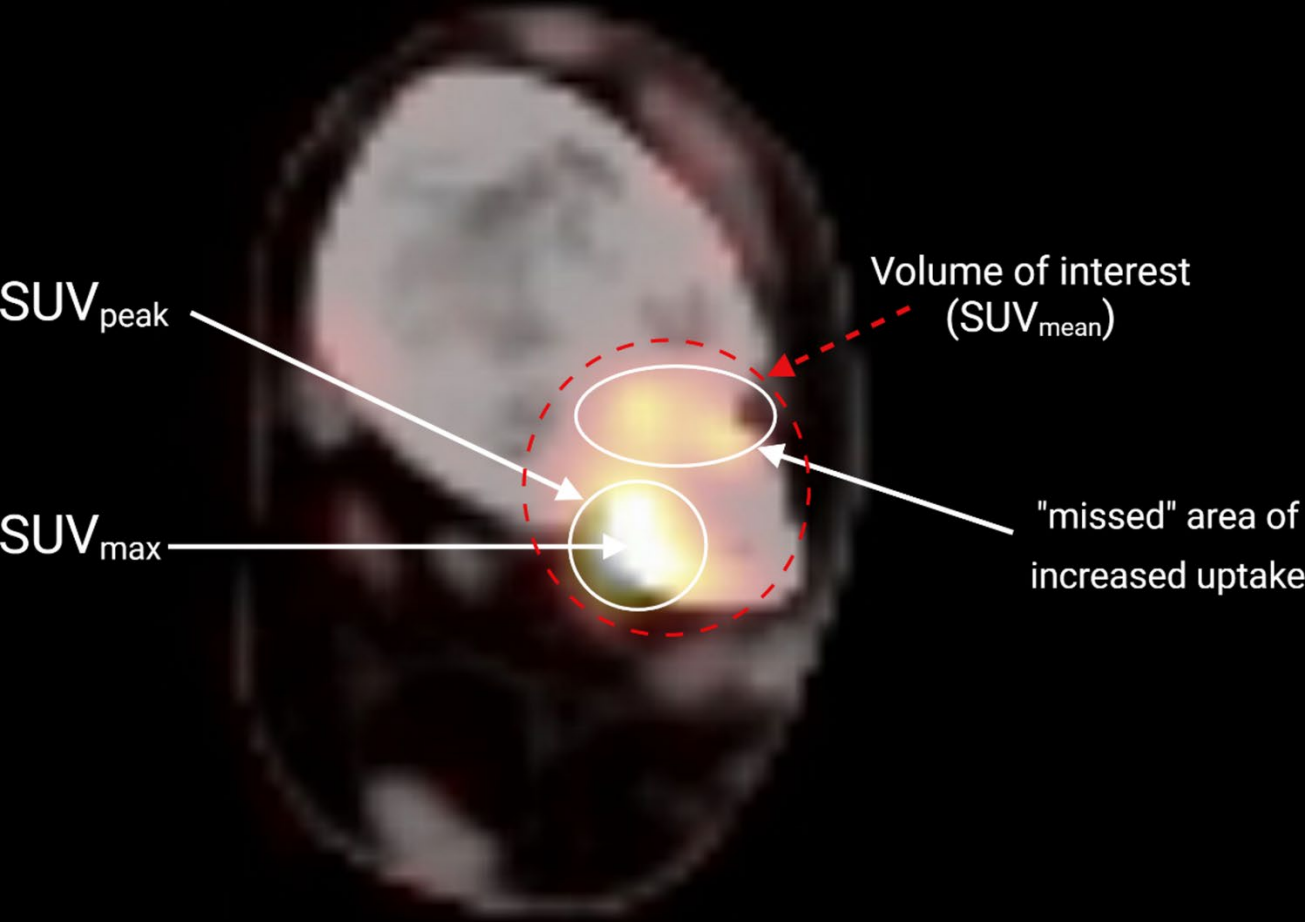
[18F]NaF PET/CT image explaining the difference between volume of interest, SUV_max_, SUV_peak_ and how an area of increased uptake can be “missed” when using SUV_max_ or SUV_peak_

This has led to the use of simplified methods as alternatives to NLR, such as the Gjedde–Patlak analysis and standardized uptake value (SUV). These methods seem to correlate well with full kinetic analysis in normal bone and have become increasingly important in quantifying skeletal ^18^F-fluoride uptake. Gjedde–Patlak analysis is also based on the compartment model, but only requires a linear regression analysis once the radiopharmaceutical tracer uptake in the target tissue from the plasma occurs at a fixed rate [8, 9]. Nevertheless, it remains difficult to perform Gjedde–Patlak analysis on a regular basis as it still requires blood sampling and a (short) period of dynamic imaging. Therefore, SUV has become the most widely used parameter for quantification in daily clinical routine [10]. SUV is a semi-quantitative measure representing tissue activity in a volume of interest (VOI) corrected for injected activity and a body anthropometric measure such as weight, lean body mass or body surface area (Fig. 1). SUV can be calculated without any blood sampling and it can be used in association with a whole-body scan enabling the measurement of tracer distribution throughout the body [11]. Note that outcome from all mentioned approached depends on the region of interest definition, and SUV can be reported in a few ways. SUV_mean_ is defined as the average SUV (and thus representing average [^18^F]NaF uptake) of all voxels within a VOI. SUV_max_ is the highest single-voxel value within a VOI, and SUV_peak_ is the average of a fixed size volume (often 1 cm^3^) centered around the hottest voxel within a VOI (Fig. 1).

In recent years, many studies have used various uptake parameters to quantify [^18^F]NaF uptake in bone disorders. The purpose of this review was to provide an overview of the parameters that presently are used to measure [^18^F]NaF uptake in bone disorders, and to assess whether they are suitable for assessing disease activity.

## Methods

A systematic search was performed in the databases: PubMed, Embase.com, and Clarivate Analytics/Web of Science Core Collection. The timeframe within the databases was from inception to 28th November 2023 and conducted by GLB and RR. The search included keywords and free text terms for (synonyms of) ‘Fluorine-18’ combined with (synonyms of) ‘positron emission’ combined with (synonyms of) ‘bone’. A full overview of the search terms per database can be found in the supplementary information (see Appendix 1). No limitations on date or language were applied in the search.

### Study selection

**Fig. 2:**
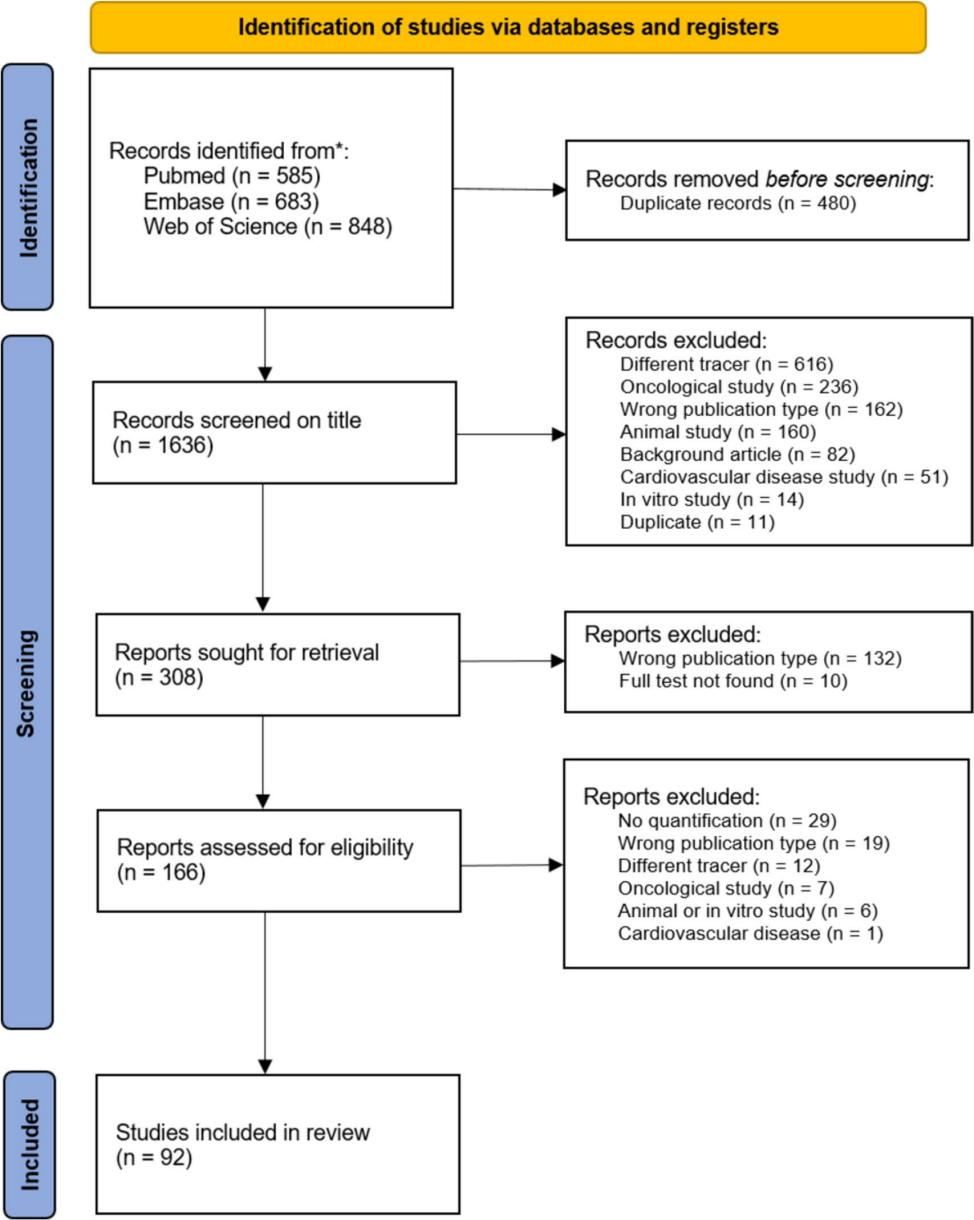
PRISMA flow diagram of the study selection

Two reviewers (RR and EE) independently screened all potentially relevant titles and abstracts for eligibility using Rayyan (web-tool to screen and select studies). Where necessary, the full text of an article was checked against the eligibility criteria. Differences in judgment were discussed and resolved by a consensus. Studies were included if they met the following criteria: (i) studies using [^18^F]NaF PET; (ii) pathological condition primarily involving bone metabolism and/or healing; (iii) [^18^F]NaF PET-derived uptake parameter reported; (iv) studies published in English; and (v) full-text availability. Studies were excluded if they concerned: (i) oncological diseases; (ii) cardiovascular diseases; (iii) studies in otherwise healthy subjects; (iv) animal and in vitro studies; and (v) certain publication types such as editorials, letters, legal cases, interviews, conference abstracts, and reviews. The PRISMA flow diagram of the study selection is shown in Fig. 2.

### Quality assessment

The full text of the selected articles was obtained for further review. Two reviewers (RDdR and EMWE) independently evaluated the methodological quality of the full-text papers using the Study Quality Assessment Tool created by NHLBI (National Heart, Lung, and Blood Institute). The results of this quality assessment can be found in Supplementary Material S2.

## Results

### Search and selection of results

The literature search generated a total of 2116 references: 584 in PubMed, 678 in Embase.com, and 623 in Clarivate Analytics/Web of Science Core Collection. After removing duplicates of references that were selected from more than one database, 1636 remained. The flow chart of the search and selection process is presented in Fig. 2.

After screening titles and abstracts, based on the selection criteria described above, 166 remained for full-text analysis. This analysis excluded a further 74 articles (Fig. 2). From the remaining 92 articles, the following data were extracted: study type, number of participants, age of participants (mean and standard deviation (SD)), quantitative parameters examined, chosen method of validation of the uptake parameters, and purpose of parameter quantification, which can be found in Table 1. In addition, details of PET methodology were extracted: PET scanner type, injected dose and scan time, reconstruction algorithm, and volume of interest (VOI) definition, which can be found in Table 2.

**Table 1:** Articles included in the review. Listed is the study type, number of participants, age of participants (mean and standard deviation (SD)), quantitative parameters examined, chosen method of validation of the uptake parameters, and purpose of parameter quantification

Author	Disease	Study type	Participants	Age (mea*n*—SD)	Parameter	Purpose and result of [18F]NaF PET parameter quantification
Kogan (2018) [83]	Anterior cruciate ligament (ACL) injury	Cross-sectional	15	32.7 (10.5)	SUV_max_	Significantly increased subchondral bone SUV_max_ and cartilage T2 times were observed in the ACL-reconstructed knees compared to the contralateral knees. Using the contralateral knee as a control, a significant correlation between the difference in subchondral bone SUV_max_ (between injured and contralateral knees) and the adjacent cartilage T2 times was observed
Jeon (2018) [37]	Ankle trauma	Cross-sectional	121	45.9 (16.7)	SUV_max_ SUV_mean_ TLF	The fracture group had higher SUV_max_, SUV_mean_, and TLF (total lesion fluorination) values than the non-fracture group. A higher SUV_max_, SUV_mean_ and TLF correlated with more limited range of motion scores in the fracture group but not in the non-fracture group
Dyke (2019) [84]	Ankle arthroplasty (total)	Prospective cohort	9	68.9 (8.2)	K1 NLR-derived K_i_ SUV_mean_	Full kinetic analysis was performed, and K_i_ was analyzed over time. K_i_ appeared to mirror the measured SUV_mean_ normalized for lean body mass, but correlation analysis was not performed. SUV_mean_ values were analyzed pre- and post-operatively in the talus, with a higher SUV_mean_ post-operatively
Bruijnen (2018) [54]	Ankylosing spondylitis	Prospective cohort	12	36.7 (10.6)	NLR-derived K_i_ SUV^AUC^ # PET positive lesions	SUV^AUC^ was the most representative semi-quantitative outcome measure for monitoring the focal tracer uptake during intervention with anti-TNF therapy, with the highest correlation with NLR-derived K_i_. Histological analysis of PET positive lesions confirmed local osteoid formation. Lesions were also followed-up on over time. After 12 weeks of anti-TNF treatment, [18F]NaF uptake in clinical responders (> 20% improvement in disease activity score (ASAS20)) decreased significantly in the costovertebral and SI joints in contrast to non-responders
Kim (2020) [32]	Ankylosing spondylitis	Prospective cohort	27	37.9 (6.2)	K1, k2, k3, k4 NLR-derived K_i_ SUV_mean_ SUV_max_	Dynamic and static parameters were independently associated with disease scores, but correlation between scores was not investigated. Response to therapy was evaluated with a disease activity score (BASDAI). NLR-derived K_i_ and SUV_mean_ were significantly different between the responders and non-responders. SUV_max_ of the spine had a significant positive correlation with BASDAI score
Lee (2020) [85]	Ankylosing spondylitis	Prospective cohort	28	35.5 (11.3)	Lesion-to-background SUV_max_ SUV_mean_	Lesion-to-background (LBR) was compared to BASDAI score and follow-up BASDAI score. The LBR of the posterior joint correlated with BASDAI score. There was no significant correlation between the other analyzed areas and follow-up BASDAI score
Bruckmann (2022) [86]	Ankylosing spondylitis	Prospective cohort	16	38.6 (12.0)	SUV_max_	Quantification of tracer uptake showed that the mean SUV_max_ for all joints in the vertebra decreased significantly upon treatment. Anti-TNF antibody treatment led to a significant decrease in uptake within 3–6 months, especially, but not solely, at sites of inflammation
Strobel (2010) [87]	Axial spondyloarthritis	Prospective cohort	28	47.0 (9.5)	SUVratio	Uptake was compared to radiographic grading of the sacroiliac joint (SIJ). Taking an SIJ/S ratio (SUV_mean_ SIJ/SUV_mean_ sacrum) of > 1.3 as the threshold, the sensitivity, specificity, and accuracy on a per patient basis were 80%, 77%, and 79%, respectively, for predicting SIJ arthritis
Brenner (2004) [38]	Bone graft healing	Prospective cohort	34	NS	NLR-derived K_i_ Patlak-derived K_i_ SUV_mean_	[18F]NaF uptake in cancellous grafts decreased by 25% from 6 to 12 months post-surgery and revealed a total decrease of 60–65% after 2 years as measured by SUV_mean_, Patlak-derived K_i_, and NLR-derived K_i_. Highly significant correlations were found between SUV_mean_, Patlak-derived K_i_, and NLR-derived K_i_ for both grafts and normal limb bones. In patients imaged repeatedly, the percentage changes in [18F]NaF also correlated significantly between SUV_mean_, Patlak-derived Ki, and NLR-derived K_i_
Pumberger (2016) [88]	Bone graft healing (spondylectomy)	Prospective cohort	8	55.7 (9.2)	SUV_max_	The SUV_max_ was 1.46 in the cage center, 8.14 in the reference vertebra, and 11.19 in adjacent endplates. Therefore, the viability of the bone within the cage was dramatically decreased compared to the reference (four-fold decreased). In contrast, the endplates showed a higher bone metabolism than the reference vertebra (1.6-fold increased)
Kobayashi (2016) [89]	Femoroacetabular impingement	Cross-sectional	27	50.0 (15.6)	SUV_max_	The SUV_max_ of in areas of impingement was significantly higher than the SUV_max_ of the contralateral regions. The SUV_max_ ratio between the affected and unaffected side correlated positively with the angle of impingement
Oishi (2019) [90]	Femoroacetabular impingement	Retrospective cohort	41	43.0 (14.4)	SUV_max_	An increased SUV_max_ corresponded with a specific femoroacetabular impingement subtype, making it possible to distinguish various morphologies of femoroacetabular impingement by measuring uptake
Botman (2019) [53]	Fibrodysplasia ossificans progressiva	Prospective cohort	5	26.9 (6.9)	SUV_peak_	SUV_peak_ over time was compared to heterotopic bone volume. A SUV_peak_ > 8.4 corresponded with an increase of heterotopic bone volume
Papadakis (2019) [20]	Fibrous dysplasia	Retrospective cohort	15	27.0 (6.9)	MAV SUV_max_ SUV_mean_ TLF	Metabolic active volume (MAV) and total lesion fluorination (TLF) significantly correlated with lifetime fractures/orthopedic/craniofacial surgeries, mean fractures/orthopedic/craniofacial surgeries per year, alkaline phosphatase (ALP), osteocalcin and n-telopeptides
van der Bruggen (2020) [18]	Fibrous dysplasia	Retrospective cohort	20	46.0 (14.1)	SUV_mean_ SUV_peak_ TLF	TLF correlated with the skeleton burden score (region-based scoring system estimating uptake per area to generate an overall disease score, SBS). TLF correlated with ALP, procollagen peptide type 1 N-terminal (P1NP), and fibroblast growth factor 23 (FGF-23). The SBS did not correlate with ALP or P1NP, but was correlated to FGF-23. SUV_peak_ did not appear to have to have any correlation of the serum biomarkers of bone metabolism
Choe (2011) [46]	Hip arthroplasty	Prospective cohort	41	72.8 (9.5)	SUV_max_	A SUV_max_ > 5.0 was indicative of sceptic loosening of the prothesis
Forrest (2006) [42]	Hip arthroplasty	Prospective cohort	15	45.0 (6.4)	SUV_mean_	The SUV_mean_ in the operated hips was significantly higher than the non-operated hips, indicating increased burn turnover. This did not differ per hip region
Kumar (2016) [45]	Hip arthroplasty	Prospective cohort	45	51.8 (15.3)	SUV_max_	A SUV_max_ > 4.4 in the early imaging phase, and SUV_max_ > 8.1 in the delayed imaging phase correlated with sceptic loosening of the implant as confirmed by surgery and histopathology
Piert (1999) [39]	Hip arthroplasty	Cross-sectional	16	71.6 (7.7)	NLR-derived K_i_ Patlak-derived K_i_	Allogenic bone grafts were characterized by a significantly increased NLR-derived K_i_ after 3–6 weeks (+ 190.9%) compared with contralateral hips but decreased almost to the baseline levels of contralateral hips (+ 45.5%) after 5 months to 9 years
Raijmakers (2014) [91]	Hip arthroplasty	Cross-sectional	22	44.8 (25.2)	NLR-derived K_i_ Patlak-derived K_i_ SUV_mean_	The Patlak-derived K_i_, for 10–60 min after injection, showed a high correlation with the NLR-derived K_i_. The highest correlation between K_i_ and lean body mass–normalized SUV was found for the interval 50–60 min. Finally, changes in SUV correlated significantly with those in NLR-derived K_i_. The present data support the use of both Patlak and SUV for assessing fluoride kinetics in humans. However, [^18^F]NaF PET has only limited accuracy in monitoring bone blood flow
Temmerman (2008) [41]	Hip arthroplasty	Prospective cohort	6	76.0 (5.0)	NLR-derived K_i_	There was a significant increase in periprosthetic bone metabolism as measured by NLR-derived K_i_ in patients in whom allogeneic bone grafts were used compared to patients where no bone grafts were used
Tezuka (2020) [92]	Hip arthroplasty	Randomized control trial	52	65.0 (12.0)	SUV_max_	The influence of a coating in hip arthroplasty in terms of bone mineral density (BMD) preservation is limited. No significant correlation was found between BMD and SUV_max_ measured by PET, either with or without the use of a hip arthroplasty in coating
Ullmark (2009) [40]	Hip arthroplasty	Prospective cohort	7	66.0 (6.4)	SUV_mean_	Uptake was 64% higher on the affected side compared to reference bone. 1 week after surgery, it was increased by 77% in segmental regions, while the uptake of the cavitary regions was at the reference level. After 4 months, the uptake was increased by 91% in cavitary regions and by 117% in segmental regions
Ullmark (2013) [43]	Hip arthroplasty	Prospective cohort	8	57.5 (7.5)	SUV_mean_	The SUV_mean_ was statistically significantly higher in both types of implants compared to the contralateral hip at 4 months and was most pronounced in the upper femur
Ullmark (2020) [44]	Hip arthroplasty	Prospective cohort	26	NS	SUV_mean_	The SUV_mean_ was 4.6 (at 6 weeks) and 3.5 (at 6 months) around the uncemented cups, and 4.8 and 4.0, respectively, for the cemented cups. Normal healthy bone metabolism in the reference bone was 2.8 and 2.7 SUV at 6 weeks and 6 months, respectively
Mechlenburg (2013) [93]	Hip dysplasia (periacetabular osteotomy)	Prospective cohort	12	36.0 (9.3)	NLR-derived K_i_	Non-linear regression fitting was not stable for the first 45 min, fitting required a scanning time of 90 min which was not obtained for most of the participants and therefore not reported
Waterval (2011) [94]	Hyperostosis cranialis interna	Prospective cohort	9	NS	SUV_max_ SUV_mean_	Uptake of patients was compared to family members acting as control patients. SUV_mean_ was significantly higher in the sphenoid bone and clivus regions of patients with hyperostosis cranialis interna
Jenkins (2017) [95]	Lower back pain	Cross-sectional	6	65.3 (10.1)	Patlak-derived K_i_ SUV_mean_ SUV_max_	Patlak-derived K_i_ correlated with disability score (and weakly with MRI facet athropathy grade). SUV_max_ and SUV_mean_ were also compared but did correlate significantly
Kobayashi (2013) [23]	Osteoarthritis (hip)	Cross-sectional	48	42.3 (15.3)	SUV_max_	Differences in the average SUV_max_ were found for each Kellgren–Lawrence (K/L) grade group. The average SUV_max_ values were increasingly higher according to K/L grade group and pain severity group
Kobayashi (2015) [30]	Osteoarthritis (hip)	Cross-sectional	43	49.8 (14.7)	SUV_max_	Joints were considered positive for PET uptake when a SUV_max_ > 6.5 was found. Most (96%) of the joints affected by osteoarthritis on the MRI were also PET positive
Kobayashi (2015) [25]	Osteoarthritis (hip)	Cross-sectional	57	50.8 (11.3)	SUV_max_	A SUV_max_ cut-off value of 7.2 (sensitivity: 1.00, specificity: 0.84) in the joint was predictive for pain worsening and 6.4 (sensitivity: 0.92, specificity: 0.83) for minimal joint space narrowing
Tibrewala (2019) [96]	Osteoarthritis (hip)	Cross-sectional	10	59.9 (12.5)	Patlak-derived K_i_ SUV_mean_ SUV_max_	Shaft thickness correlated with SUV_mean_ and SUV_max_ in the femur and Patlak-derived K_i_ in the acetabulum. Pain had increased correlations with SUV_mean_ and SUV_max_ in the acetabulum and femur when shaft thickness was considered
Yellanki (2018) [25, 26]	Osteoarthritis (hip)	Retrospective cohort	116	48.6 (10.7)	SUV_mean_	SUV_mean_ for the hip positively correlated with BMI as a risk factor for developing osteoarthritis, but not to age
Jena (2022) [24]	Osteoarthritis (knee)	Cross-sectional	16	42.7 (11.8)	SUV_max_	Globally the mean SUV_max_ was found to increase according to K/L score. There is a proportional increase in SUV_max_ with the size of osteophytes
Jena (2023) [29]	Osteoarthritis (knee)	Cross-sectional	16	41.0 (11.8)	SUV_max_	Bone marrow lesions and osteophytes with a higher MRI osteoarthritis knee score (MOAKS) score showed higher SUV_max_
Mackay (2021) [97]	Osteoarthritis (knee)	Cross-sectional	11	54.0 (12.0)	SUV_max_ K_trans_	SUV_max_ was positively associated with adjacent K_trans_ (the volume transfer coefficient between the blood plasma and the extracellular extravascular space). Synovitis is more intense adjacent to peripheral bone regions with increased metabolic activity than those without, although there is some overlap. Subregional bone metabolic activity is positively associated with intensity of adjacent synovitis
Savic (2016) [28]	Osteoarthritis (knee)	Prospective cohort	16	NS	Patlak-derived K_i_ SUV_mean_ SUV_max_	SUV_mean_ correlated highly with Patlak-derived K_i_. Degenerative changes on the MRI were associated with increased bone turnover on the PET. Associations between pain and increased bone uptake were seen in the absence of morphological lesions in cartilage, but the relationship was reversed in the presence of incident cartilage lesions
Tibrewala (2020) [27]	Osteoarthritis (knee)	Prospective cohort	29	55.9 (8.6)	SUV_mean_	Using the mean values of the MRI intensity and SUV of all the patients in the various VOIs in a regression model were able to predict the bone and cartilage lesion scores as measured by K/L and WORMS scores
Watkins (2021) [98]	Osteoarthritis (knee)	Prospective cohort	31	65.6 (10.4)	K1 NLR-derived K_i_ SUV_max_	Mean and maximum SUV and kinetic parameters K_i_, K1, and extraction fraction were significantly different in the knee joint between healthy subjects and subjects with osteoarthritis. Between-group differences in metabolic parameters were observed both in regions where the osteoarthritis group had degenerative changes as well as in regions that appeared structurally normal. Uptake parameters were not correlated to each other
Watkins (2022) [99]	Osteoarthritis (knee)	Prospective cohort	10	59.0 (8.0)	K1 NLR-derived K_i_ SUV_mean_, SUV_max_	There was a significant increase in [^18^F]NaF uptake as measured in NLR-derived K_i_, K1, SUV_mean_ and SUV_max_ in osteoarthritis exercised knees, differing per bone region
Christersson (2018) [58]	Osteomyelitis	Prospective cohort	8	52.0 (11.0)	SUV_max_ SUV_max_ ratio	[^18^F]NaF uptake and [^18^F]FDG uptake were evaluated in areas suspicious for osteomyelitis. [^18^F]NaF SUV_max_ compared to contralateral regions and was found to be elevated in infected areas as confirmed with tissue histology. SUV_max_ between [^18^F]NaF and [^18^F]FDG correlated highly. Combination of tracers leadS to better identification of area requiring resection during surgery
Freesmeyer (2014) [56]	Osteomyelitis	Cross-sectional	11	53.0 (6.9)	SUV_max_ SUV_mean_	The early dynamic sequence SUV values correlated with SUV values obtained from the later static sweep. Uptake was compared to diagnosis already made on other radiographical images. The affected bone area showed significantly higher SUV_max_ and SUV_mean_ compared to the healthy contralateral region. The affected bone areas also significantly differed from non-affected contralateral regions in conventional late [^18^F]NaF PET/CT
Reinert (2022) [57]	Osteomyelitis (jaw)	Prospective cohort	6	55.3 (10.0)	SUV_mean_	SUV_mean_ in affected jawbone was significantly increased in all patients compared to healthy jawbone
Kubota (2015) [64]	Osteonecrosis (femoral head)	Cross-sectional	42	39.5 (11.0)	SUV_max_	SUV_max_ increased according to the progression of the Ficat classification stage. The mean SUV_max_ was significantly higher in the collapse group than the non-collapse group (P < 0.01). The cut-off SUV_max_ of 6.45 (sensitivity: 0.80, specificity: 0.92) was used for the prediction of femoral head collapse
Aratake (2009) [65]	Osteonecrosis (knee)	Prospective cohort	13	70.0 (5.0)	SUV_max_	The SUV_max_ was measured at different disease stages (SONK). There were no significant differences in these measurements between the SONK stages. However, a significant positive correlation between the SUVmax and lesion size, including the surface area of the lesion and the condyle width ratio, was found. The approximate volumes of the lesions also showed a significant correlation with the SUV_max_
Schiepers (1998) [66]	Osteonecrosis (femoral head)	Cross-sectional	5	33.0 (9.2)	NLR-derived K_i_	The femoral head affected by osteonecrosis exhibited lower uptake as measured by NLR-derived K_i_ than femoral heads unaffected
Frost (2008) [71]	Osteoporosis	Cross-sectional	16	64.3 (6.6)	NLR-derived Ki Patlak-derived Ki SUV_mean_	The precision of the PET parameters ranged from 12.2% for K_i_−3 k to 26.6% for K_i_−4 k. The individual precision errors in K_i_ for each subject were significantly greater using the 2t4k model than the 2t3k model or Patlak model. No significant difference in precision was found among K_i_−2t3k, K_i_-Patlak, and SUV_mean_. The precision values of the 3 biochemical markers were similar to the value observed for K_i_ using the 2t3k model and Patlak and SUV_mean_ but were less than that observed using the 2t4k model. Direct comparison of individual [^18^F]NaF uptake and biochemical markers was not performed
Jassel (2019) [67]	Osteoporosis	Retrospective cohort	63	NS	NLR-derived K_i_ SUV_mean_	Correlation between SUV, hounsfield units (HU), bone mineral apparent density (BMAD), bone mineral density (BMD) was analyzed. Uptake as measured by NLR-derived K_i_ correlated positively with HU, BMAD, and BMD. Correlations were highest between NLR-derived K_i_ and HU and lowest between NLR-derived K_i_ and areal BMD. Performance of SUV_mean_ was comparable to NLR-derived K_i_
Park (2023) [100]	Osteoporosis	Retrospective cross-sectional	88	48.0 (15.6)	SUV_mean_	There was a significant negative correlation between SUV_mean_ and age in females and a weaker, but also significant correlation in males
Puri (2021) [101]	Osteoporosis	Cross-sectional	30	61.0 (5.8)	NLR-derived K_i_ at different time intervals	A comparison was performed for 2t3k-k_i_ performance at different time points to investigate whether similar K_i_ measurements could be found using a shorter time interval. K_i_ measurements with statistical power equivalent or superior to conventionally analyzed 60-min dynamic scans were obtained with scan times as short as 12 min
Rhodes (2020) [102]	Osteoporosis	Retrospective cohort	139	52.0 (15.6)	Bone metabolism score (BMS) SUV_mean_	Age was negatively correlated with left and right femoral head BMS (SUV of bone exceeding 100 HU/SUV of total region * 100), predominately in the cortical bone. BMD was positively correlated with whole and cortical BMS
Uchida (2009) [103]	Osteoporosis (Alendronate treatment)	Cross-sectional	24	59.6 (5.4)	SUV_mean_	Lumbar spine SUV_mean_ measurements were significantly lower in the osteoporotic group (T-score ≤ − 2.5) than in the group that was healthy or osteopenic (T-score > − 2.5). Although there was a significant correlation between BMD and SUV in the lumbar spine at baseline, there was no correlation between the 2 variables at 12 months of treatment with alendronate
Frost (2003) [104]	Osteoporosis (Risendronate treatment)	Prospective cohort	18	67.0 (4.6)	K1, k2, k3, k4, k3/[k2 + k3] NLR-derived K_i,_	Mean vertebral K_i_ decreased significantly by 18.4% from baseline to 6 months post-treatment. This decrease was similar in magnitude to the decrease observed for ALP
Frost (2011) [68]	Osteoporosis (Teriparatide treatment)	Cross-sectional	20	65.3 (8.2)	NLR-derived Ki, K1, k2, k3, k4 SUV_mean_	Change in NLR-derived K_i_ in the spine was compared to changes in BTMs after 6 months of teriparatide therapy. None of the four correlations were statistically significant. Change in NLR-derived K_i_ in the lumbar vertebral bodies showed a highly significant change from baseline with a mean percentage increase of 23.8%. This correlated poorly with SUV measurement in the spine
Siddique (2011) [105]	Osteoporosis (Teriparatide treatment)	Cross-sectional	40	65.3 (8.2)	NLR-derived K_i_ (2t3k and 2t4k model) Patlak-derived K_i_ SUV_mean_	Methods that calculated K_i_ assuming K4 = 0 required fewer subjects to demonstrate a statistically significant response to treatment than methods that fitted K4 as a free variable. Although SUV gave the smallest precision error, the absence of any significant changes makes it unsuitable for examining response to treatment in this study
Fushimi (2022) [106]	Osteoporosis (Zolendronic acid and denosumab treatment)	Matched, case–control	23	70.2 (10.8)	SUV_mean_	The mean SUVs of the thoracic vertebrae in the denosumab and control group were not significantly different. The mean SUV of the cervical vertebrae in the zolendronic acid group were significantly lower than that in the control group
Puri (2012) [69]	Osteoporosis (postmenopausal bone turnover)	Retrospective cross-sectional	12	61.5 (5.5)	NLR-derived K_i_ (2t3k and 2t4k model) Patlak-derived K_i_ SUV_mean_	Correlations between 2t4k-K_i_ and 2t3k-K_i_, Patlak-derived K_i_ and SUV measured in the hip and lumbar spine combined were high with correlations of 0.91, 0.97, and 0.93, respectively
Cook (2000) [21]	Osteoporosis (postmenopausal bone turnover)	Cross-sectional	26	62.0 (8.8)	K1, k2, k3, k4 NLR-derived K_i_	Mean vertebral K_i_ and k1 in the lumbar vertebrae were found to be significantly greater than K_i_ and k1 in the humerus but no significant differences were found in K2, K3, and K4
Frost (2004) [107]	Osteoporosis (postmenopausal bone turnover)	Cross-sectional	72	61.0 (7.9)	k3/(k2 + k3) NLR-derived K_i_	Vertebral estimates of NLR-derived K_i_ were significantly lower in the osteoporotic group compared with both the osteopenic and normal groups. A significant positive correlation was observed between BMD and K_i_ and the fraction of the tracer that bound to the bone mineral [k3/(k2 + k3). A significant negative correlation was observed between levels of ALP and the fraction of tracer that bound to bone mineral
Frost (2009) [108]	Osteoporosis (postmenopausal bone turnover)	Cross-sectional	23	64.3 (4.4)	K1, k2, k3, k4, Ki/k1 NLR-derived K_i_	Mean bone perfusion K1 and bone turnover K_i_ were significantly higher at the lumbar spine compared to the humerus for both treatment-naïve and antiresorptive groups
Puri (2013) [70]	Osteoporosis (postmenopausal bone turnover)	Cross-sectional	12	62.6 (5.3)	K1 NLR-derived K_i_ SUV_mean_	Values of K1, NLR-derived K_i_ and SUV at the femoral neck and femoral shaft were three times lower than at the lumbar spine. Among the proximal femur sites, NLR-derived K_i_ and SUV were lower at the femoral shift compared with the femoral neck. Spearman correlation coefficient between K1, NLR-derived K_i_ and SUV_mean_ was highly statistically significant
Cook (2002) [109]	Paget’s disease	Prospective cohort	7	70.7 (ns)	K1, k2, k3, k4 K1/k2, K_i_/K1 NLR-derived K_i_	Compared with normal bone, pagetic bone demonstrated higher values of NLR-derived K_i_ and k1, reflecting increased mineralization and blood flow, respectively. A high correlation was found between ALP levels and K_i_ in pagetic bone
Installe (2015) [22]	Paget’s diseases	Cross-sectional	14	64.8 (12.7)	K1, K_i_/K1 NLR-derived K_i_ Patlak-derived K_i_ SUVmax	Baseline uptake of [^18^F]NaF by pagetic bones was significantly higher than in normal bones. SUV_max_ correlated with both Patlak-derived K_i_ and NLR-derived K_i_ at baseline, 1 month, and 6 months. Moreover, the change of SUV_max_ between baseline and 1 month, as well as between baseline and 6 months, also correlated with the change of Patlak-derived K_i_ and NLR-derived K_i_
Waterval (2013) [35]	Otosclerosis	Prospective cohort	21	59.5 (12.7)	SUV_max_ SUV_mean_	Against grading on CT and hear loss, the relation between CT otosclerosis classification and SUV_mean_ values at different anatomical subsites was investigated; a significant correlation was found at the saccule between these two. Significant correlations between audiogram classification and SUV_mean_ values were present for the fenestral and saccule areas and the posterior part of the internal auditory canal
Draper (2012) [36]	Patellofemoral pain	Cross-sectional	20	31.0 (5.2)	SUV_mean_ SUV_peak_	SUV was compared against experienced pain as evaluated by standardized pain questionnaires. Patients with painful knees exhibited increased tracer uptake compared to the pain-free knees of four subjects with unilateral pain and there was a correlation between increasing SUV_peak_ and pain intensity
Graf (2020) [74]	Primary hyperparathyroidism and brown tumors	Retrospective cohort	8	49.3 (13.6)	MAV	MAV was correlated with PTH and ALP, and the requirement for intense post-operative calcium substitution, which determines the duration of hospitalization. The total MAV of the brown tumor per patient correlated positively with serum calcium. MAV correlated significantly with serum PTH, ALP and duration of post-operative hospitalization
Hochreiter (2019) [110]	Reverse shoulder arthroplasty	Retrospective cohort	7	84.5 (3.8)	SUV_max_	The mean value of SUV_max_ of the allografts was compared to the reference vertebrae but was not statistically different, implying viability and fusion in all allografts
Jonnakuti (2018) [111]	Rheumatoid arthritis (knee arthrosis)	Cross-sectional	18	56.7 (12.4)	SUV_mean_ TBR	SUV correlated with K/L score. Unadjusted global SUV_mean_ of the knee or femoral neck scores did not significantly correlate with average K/L grading. Higher TBR scores of the knee were observed among individuals with higher average K/L grading scores
Park (2021) [33]	Rheumatoid arthritis	Cross-sectional	17	53.8 (9.5)	SUV_max_ TBR	Tender and swollen joints had a significantly higher SUV_max_ and joint-to-bone uptake ratio than joints without synovitis. On correlation analysis, summed joint SUV_max_ and summed joint-to-bone uptake ratio of 28 joints showed strong positive correlation with a rheumatoid arthritis disease score (DAS28-ESR). The summation of both PET/CT parameters of 28 joints showed a diagnostic accuracy of 100.0% for predicting high disease activity in rheumatoid arthritis
Reddy (2023) [112]	Rheumatoid arthritis	Cross-sectional	18	57.3 (11.9)	SUV_mean_	In the knees, SUV_mean_ significantly correlated with body weight, BMI, leptin, and sclerostin levels. No significant correlation was observed between either PET parameter and age, height, erythrocyte sedimentation rate, and interleukins 1 and 6
Watanabe (2016) [34]	Rheumatoid arthritis	Prospective cohort	12	60.0 (11.8)	SUV_max_	SUV_max_ was compared against radiographic erosion on X-ray and estimated yearly progression of total radiographic scores. Progression significantly correlated with the SUV_max_. DAS28 and physical function assessments were also performed but not compared to [18F]NaF uptake
Aaltonen (2020) [14]	Renal osteodystrophy	Cross-sectional	26	63.0 (13.3)	Fractional uptake rate Patlak-derived K_i_	Fractional uptake rate (FUR) was calculated with Patlak-derived K_i_ by dividing Patlak-derived K_i_ in the area of interest by the AUC blood activity. There was a statistically significant correlation between mean Patlak-derived Ki and FUR levels and a majority of the histomorphometric parameters, such as bone formation rate, activation frequency, mineralized surface per bone surface and osteoblast- and osteoclast surfaces. There was also a statistically significant correlation between osteoid thickness and fluoride activity at the anterior iliac crest measured with FUR_mean_. However, there was no correlation between mean K_i_ and FUR_mean_ and osteoid volume of bone volume or mineralization lag time. There was a statistically significant correlation between PTH and K_i_ mean and FUR_mean_ levels. There was no statistical correlation between K_i_ mean and FUR_mean_ and ionized calcium and ALP. A weak correlation between phosphorus and K_i_ and FUR_mean_ was also observed
Aaltonen (2021) [13]	Renal osteodystrophy	Cross-sectional	26	63.0 (13.3)	Fractional uptake rate Patlak-derived K_i_	K_i_ and FUR were compared to histologic classification of renal osteodystrophy (ROD) (high/mild/low). In ROC analysis for discriminating high turnover/hyperparathyroid bone disease from other types of ROD, using unified TMV-based classification, K_i_ cut-off > 0.055 Ml/min/Ml in the PET scan had an AUC of 0.86, the sensitivity was 82% and specificity 100%, the negative predictive value 88% and positive predictive value 100%. In ROC analysis for discriminating low turnover/adynamic bone disease from other types of ROD, using unified TMV-based classification, fluoride activity cut-off < 0.038 Ml/min/Ml in the PET scan had an AUC of 0.87 with 100% sensitivity and 70% specificity, the negative predictive value was 100% and positive predictive value
Frost (2013) [17]	Renal osteodystrophy	Cross-sectional	19	64.0 (15.4)	NLR-derived K_i_ NLR-derived K_i_/BMAD	Significant differences in NLR-derived K_i_ between different skeletal sites were observed for both the CKD stage 5 and osteoporosis groups. NLR-derived K_i_ was also compared to bone mineral density as measured on the DXA scan. Significant correlation between K_i_/BMAD and mineral acquisition apposition rate in histological analysis was observed but not between K_i_/BMAD and other histological mineral density parameters
Fuglo (2023) [113]	Renal osteodystrophy	Cross-sectional	10	67.0 (5.8)	Patlak-derived K_i_ NLR-derived K_i_	Various input functions toward calculating NLR-derived K_i_ and Patlak-derived K_i_ were compared. The NLR-derived K_i_ from the femoral bone VOI’s correlated positively to PTH and showed significant differences between patients and controls
Vrist (2021) [15]	Renal osteodystrophy	Prospective cohort	17	62.5 (10.1)	Patlak-derived K_i_ NLR-derived K_i_	K_i_ from Patlak analyses correlated well with non-linear regression analysis. NLR-derived K_i_ correlated with bone turnover parameters obtained through bone biopsy, being able to detect a low bone turnover with a high sensitivity (83%) and specificity (100%)
Vrist (2022) [16]	Renal osteodystrophy	Prospective cohort	17	62.5 (10.1)	Estimation of Patlak-derived K_i_ from static images	The pelvic K_i_ from [18F]NaF PET/CT correlated with bone turnover parameters obtained by bone biopsy. CT-derived radiodensity correlated with bone volume. Of the biomarkers, only osteocalcin showed a correlation with turnover assessed by histomorphometry
Constantinescu (2020) [50]	Spinal interbody fusion (posterior lumbar interbody fusion)	Prospective cohort	18	67.8 (5.2)	SUV	SUV was compared for fused and unfused patients, though these did not differ. The [18F]NaF uptake did not correlate with the chronological change in the clinical parameters
Peters (2015) [52]	Spinal interbody fusion (posterior lumbar interbody fusion)	Prospective cohort	36	NS	SUV_max_	SUV_max_ in the vertebral endplates was significantly higher in patients in the lowest Oswestry Disability Index category (i.e., with the worst clinical performance) than in patients in higher categories. The visual analog scale and EuroQol results were similar although less pronounced, with only SUV_max_ between category 1 and 2 being significantly different
Peters (2015) [51]	Spinal interbody fusion (posterior lumbar interbody fusion)	Prospective cohort	16	NS	NLR-derived K_i_ Patlak-derived K_i_ K1, k2, k3, Vb K1/k2 k3/(k2 + k3) SUV_max_ SUV_mean_	Statistically significant differences between control and operated regions were observed for SUV_max_, SUV_mean_, NLR-derived Ki, Patlak-derived K_i_, K1/k2 and k3/(k2 + k3). Diagnostic CT showed pseudarthrosis in 6/16 patients, while in 10/16 patients, segments were fused. Of all parameters, only those regarding the incorporation of bone (NLR-derived K_i_, Patlak-derived Ki, k3/(k2 + k3)) differed statistically significantly in the intervertebral disc space between the pseudarthrosis and fused patients group
El Yaagoubi (2022) [49]	Spinal Fusion (aseptic pseudoarthrosis)	Retrospective cohort	18	58.5 (14.7)	SUV_max_ SUV_ratio_	Statistically significant difference in SUV_max_ values (around cage/intervertebral disk space) and uptake ratios between the revision surgery and control groups. An increased SUV_max_ was also indicative of aseptic pseudoarthrosis
Lee (2019) [55]	Surgical site infection	Retrospective cohort	23	NS	Lesion-to-blood ratio Lesion-to-bone ratio Lesion-to-muscle ratio SUV_max_ SUV_mean_	Diagnosis was made against other markers (clinical, microbiological or radiographical). Lesion-to-blood pool uptake ratio on early phase scan showed the highest area under the receiver operating characteristic curve value with the cut-off value of 0.88 showing sensitivity, specificity, and accuracy of 85.7%, 88.9%, and 87.0%, respectively
Lee (2013) [63]	Temporomandibular joint disorder	Cross-sectional	24	32.0 (14.0)	SUVmean, TMJ-to-skull ratio, TMJ-to-spine ratio TMJ-to-muscle ratio	Temporomandibular joint disorder (TMD) with osteoarthritis had a high temporomandibular joint (TMJ) uptake ratio on 18F-PET/CT. The TMJ-to-skull uptake ratio on PET/CT showed the highest sensitivity (89%) and accuracy (81%) of all the uptake ratios examined
Suh (2018) [62]	Temporomandibular joint disorder	Prospective cohort	76	40.3 (17.1)	SUV_max_	Uptake was compared against disease activity and symptoms. SUV_max_ was significantly greater in arthralgic TMJs than in non-arthralgic TMJs. SUV_max_ was also significantly greater in TMJ osteoarthritis than in non-TMJ osteoarthritis and asymptomatic TMJs
Lundblad (2017) [47]	Tibia bone healing (complex fractures, osteomyelitis, osteotomies)	Prospective cohort	24	46.3 (17.6)	SUV_max_ SUV_mean_ SUV_mean_ change per day	Uptake was evaluated in the fracture healing process. SUV_mean_ and SUV_max_ difference per day did not appear to have a consistent pattern throughout the bone-healing progress. Dynamic analysis was performed and compared to simplified parameters but not reported
Lundblad (2015) [114]	Tibia bone healing (with Taylor Spatial Frame)	Prospective cohort	18	42.5 (14.4)	Patlak-derived K_i_ SUV_mean_ SUV_max_	Correlation analysis was performed of SUV_mean_ against Patlak K_i_ (r = 0.92). Fracture healing region compared to reference bone and muscle. The site of the fracture showed increased uptake in the Patlak-derived Ki, compared to reference muscle and bone on a per patient basis, though no statistical analysis was performed
Sanchez-Crespo (2017) [48]	Tibia bone healing (with Taylor Spatial Frame)	Prospective cohort	24	45.2 (17.0)	NLR-derived Ki, SUVmax	NLR-derived Ki and SUVmax correlated poorly to each other. NLR-derived K_i_ differed significantly between the separate orthopedic conditions (pseudoarthrosis, deformity, fracture)
Rauscher (2015) [115]	Unclear foot pain	Prospective cohort	22	41.0 (13.3)	SUV_mean_ SUV_max_	Multiple pathologies (osteoarthritis and stress fractures) were analyzed and determined on MRI and CT images. Increased 18F-fluoride correlated with a concurrent radiological diagnosis for SUV_mean_ and SUV_max_
Lima (2018) [61]	Unilateral condylar hyperplasia (UCH)	Prospective cohort	20	26.1 (8.1)	SUV_max_ SUV_ratio_	SUV_max_ measured in the affected condyle was significantly higher than in the unaffected condyle
Ahmed (2016) [60]	Unilateral condylar hyperplasia	Prospective cohort	16	19.5 (2.6)	SUV_max_	The affected condyle was compared to the unaffected condyle. A statistically significant difference was present between the mean percentage difference of SUV_max_ of the affected and unaffected samples
Saridin (2009) [59]	Unilateral condylar hyperplasia	Prospective cohort	13	28.0 (7.5)	NLR-derived Ki	No evidence of an abnormally high rate of bone growth in the affected condylar region in UCH patients. Instead, the rate of bone growth appeared to be reduced in the contralateral condylar region
Schiepers (1997) [116]	Various bone disorders	Cross-sectional	9	64.0 (7.5)	NLR-derived Ki	Metabolically active zones have an increased influx rate and permits classification of bone disorders and can in potential monitor therapy response in metabolic bone disease

**Table 2:** PET methodology details

Authors	Disease	PET scanner	PET/CT/MRI	Scan time and injected dose	Reconstruction algorithm details reported	VOI method
Kogan (2018) [83]	Anterior cruciate ligament (ACL) injury	PET–MR hybrid system (GE SIGNA, GE Healthcare, Milwaukee, WI)	PET/MRI	Dose: 74–111 MBq Scan time: static sweep 45 min after injection	Matrix: 256 × 128 MR-based attenuation correction	Manual On MRI and PET images separately Based on anatomical boundaries on the MRI and hot spots on the PET images
Jeon (2019)	Ankle trauma	Biograph mCT (Siemens Healthcare, Munich, Germany)	PET/CT	Dose: 5.18 MBq/kg Scan time: static sweep 60 min after injection	NS	NS
Dyke (2019) [84]	Ankle arthroplasty (total)	Biograph 64-slice Siemens mCT (Siemens Medical Systems, Knoxville, TN, USA)	PET/CT	Dose: 185 – 370 mBq Scan time: Dynamic sequence (4 × 15 s, 5 × 30 s, 2 × 60 s, 2 × 120 s, 4 × 240 s, and 3 × 300 s) totaling 45 min. Data were summed to create the static image set	NS	Manual On CT images Based on anatomical boundaries
Bruijnen (2018) [54]	Ankylosing spondylitis	Gemini TF or Ingenuity TF (Philips Healthcare, Andover, MA, USA)	PET/CT	Dose: 111 MBq Scan time: 30 min dynamic sequence, 45 min post-injection the whole-body static sweep was performed	NS	Manual On PET images
Kim (2020) [32]	Ankylosing spondylitis	Gemini (Philips Healthcare, Andover, MA, USA)	PET/CT	Dose: 322 – 396 MBq Scan time: 30 min dynamic sequence, immediately followed by a static sweep	NS	Unclear, erroneous referencing in the article
Lee (2020) [85]	Ankylosing spondylitis	Biograph 6 (Siemens Medical Systems, Knoxville, TN, USA)	PET/CT	Dose: 185 MBq Scan time: 60 min after injection	Matrix: 168 × 168 Algorithm: a standard iterative algorithm	Manual On PET images
Bruckmann (2022) [86]	Ankylosing spondylitis	Biograph mMR (Siemens Medical Systems, Knoxville, TN, USA)	PET/MRI	Dose: 161 ± 8 MBq Scan time: 40 min after injection	Correction: attenuation, truncation	Manual On MRI images
Strobel (2010) [87]	Axial spondyloarthritis	Discovery STE or Discovery Rx (GE Health Systems, Milwaukee, WI)	PET/CT	Dose: 100 – 150 MBq Scan time: 30 – 45 min after injection	Algorithm: OSEM	Manual On CT images
Brenner (2004) [38]	Bone graft healing	Advance Tomograph (General Electric Medical Systems, Waukesha, WI)	PET	Dose: 3.7 MBq/kg Scan time: 60 min dynamic sequence (4 × 20 s 4 × 40 s, 4 × 60 s, 4 × 180 s, 8 × 300 s)	Matrix: 128 × 128 Algorithm: filtered back-projection using a Hanning filter Correction: Random and scattered coincidences, attenuation and decay	Manual with a range of fixed-sized VOIs On PET images Based on anatomical boundaries
Pumberger (2016) [88]	Bone graft healing (spondylectomy)	Gemini TF 16 PET/CT system (Philips Healthcare, Andover, MA, USA)	PET/CT	Dose: 200 MBq Scan time: 45 min after injection	Correction: attenuation	Manual On PET/CT (fused) images
Kobayashi (2016) [89]	Femoroacetabular impingement	SET-2400W instrument (Shimadzu, Kyoto, Japan)	PET	Dose: 185 MBq Scan time: 40 min after injection	Matrix: 128 × 128 Algorithm: OSEM Correction: attenuation	Manual On PET images Based on anatomical boundaries (overlaid on separate acquired CT and MRI images)
Oishi (2019) [90]	Femoroacetabular impingement	Celesteion (Toshiba Medical Systems Corporation, Tochigi, Japan)	PET/CT	Dose: 185 MBq Scan time: 40 min after injection	NS	Manual On CT images
Botman (2019) [53]	Fibrodysplasia ossificans progressiva	Gemini TF-64 (Philips Medical Systems, Best, The Netherlands)	PET/CT	NS	NS	Manual On CT images Based on a threshold of 80 HU and anatomical boundaries
Papadakis (2019) (20)	Fibrous dysplasia	NS	NS	NS	NS	NS
van der Bruggen (2019) [18]	Fibrous dysplasia	Philips Gemini TF TOF 64 T (Philips Healthcare; Eindhoven, The Netherlands) GE Discovery MI (GE Healthcare; Chicago, Illinois)	PET/CT	Dose: 1.00 MBq/kg (0.93–1.06 MBq/kg) Scan time: static sweep 49 min (44–67 min) after injection	NS	Manual and semi-automatic On CT and PET images Based on a SUV cut-off for PET activity
Choe (2011) [46]	Hip arthroplasty	SET 2400 W machine (Shimadzu, Kyoto, Japan)	PET	Dose: 185 MBq Scan time: static sweep 40 min after injection	Matrix: 128 × 128 Algorithm: OSEM Correction: attenuation	NS
Forrest (2006) [42]	Hip arthroplasty	Siemens ECAT EXACT-31 PET scanner (Siemens Medical Systems, Knoxville, TN, USA)	PET	Dose: 250 MBq Scan time: static sweep 45 min after injection	Matrix: 128 × 128 Algorithm: filtered back-projection algorithm with a Hanning filter Correction: attenuation, scattered and random coincidences	Manual On PET images Based on anatomical boundaries
Kumar (2016) [45]	Hip arthroplasty	Biograph 64 (Siemens Medical Solutions, Erlangen, Germany)	PET/CT	Dose: 150 – 180 MBq Scan time: an early blood pool phase and delayed uptake phase images were acquired immediately and 20–30 min	Algorithm: OSEM	Manual On PET images Based on observed PET activity
Piert (1999) [39]	Hip arthroplasty	Advance PET scanner (General Electric Medical Systems, Milwaukee, Wisconsin)	PET	Dose: 370 MBq Scan time: 60 min dynamic sequence (12 × 10 s, 6 × 30 s, 5 × 300 s, 3 × 600 s)	NS	Manual On CT images Input function was derived from arterial samples out of the A. Radialis
Raijmakers (2014) [91]	Hip arthroplasty	EXACT HR + scanner (Siemens Medical Systems, Knoxville, TN, USA)	PET	Dose: 100 MBq Scan time: 60 min dynamic sequence (6 × 5 s, 6 × 10 s, 3 × 20 s, 5 × 30 s, 5 × 60 s, 8 × 150 s, and 6 × 300 s)	Matrix: 128 × 128 Algorithm: filtered back-projection with a Hanning filter Correction: decay, scatter, randoms, and (measured) photon attenuation	Manual On PET images Based on anatomical regions
Temmerman (2008) [41]	Hip arthroplasty	EXACT HR + scanner (Siemens Medical Systems, Knoxville, TN, USA)	PET	Dose: 100 MBq Scan time: 60 min dynamic sequence (6 × 5 s, 6 × 10 s, 3 × 20 s, 5 × 30 s, 5 × 60 s, 8 × 150 s, and 6 × 300 s)	Matrix: 128 × 128 Algorithm: filtered back-projection with a Hanning filter Correction: decay, scatter, randoms, and (measured) photon attenuation	Manual On PET images Based on anatomical regions
Tezuka (2020) [92]	Hip arthroplasty	Celesteion^™^ scanner (Toshiba Medical Systems Corporation, Tochigi, Japan)	PET/CT	Dose: 185 MBq Scan time: static sweep 45 min after injection	NS	Manual On CT images According to pre-existing radiographical areas (Gruenn zones)
Ullmark (2009) [40]	Hip arthroplasty	Siemens/CTI Exact HR + scanner (Siemens/CTI, Knoxville, TN)	PET/CT	Dose: 200 MBq Scan time: static sweep 30 min after injection	Algorithm: filtered back-projection Correction: attenuation, scatter, and decay	NS
Ullmark (2013) [43]	Hip arthroplasty	Discovery ST (General Electrics, Milwaukee, Tennessee)	PET/CT	Dose: 200 MBq Scan time: static sweep 30 min after injection	Algorithm: filtered back-projection Correction: attenuation, scatter, and decay	Semi-automatic According to a polar map method dividing the hip into various separate regions
Ullmark (2020) [44]	Hip arthroplasty	Discovery ST (General Electrics, Milwaukee, Tennessee)	PET/CT	Dose: 140 MBq Scan time: static sweep 40 min after injection	Algorithm: OSEM with 2 iterations and 21 subsets Correction: attenuation, scatter, and decay	Semi-automatic According to a polar map method dividing the hip into various separate regions
Mechlenburg (2013) [93]	Hip dysplasia (periacetabular osteotomy)	Biograph 40 (Siemens Healthcare, Knoxville, TN) Biograph 40 Truepoint (Siemens Healthcare, Knoxville, TN)	PET/CT	Dose: NS Scan time: dynamic sequence of 90 min. Plasma IF was obtained via forty arterial blood samples	Matrix: 128 × 128 Corrections: random events, detector sensitivity, dead time, attenuation, and scatter	Manual On CT images Based on anatomical boundaries
Waterval (2010)	Hyperostosis cranialis interna	Siemens Biograph mCT-4R 64 slice (Siemens Medical Solutions USA, Inc., Malvern, PA, USA)	PET/CT	Dose: 150 MBq Scan time: 60 min after injection	Matrix: 256 × 256 with a Gaussian filter of 5 mm Algorithm: filtered back-projection	Manual On CT images Based on anatomical boundaries
Jenkins (2017)	Lower back pain	3 T Signa PET/MR imaging scanner (GE Healthcare, Milwaukee, Wisconsin)	PET/MRI	Dose: 170.2 ± 29.6 MBq Scan time: 60 min dynamic sequence (12 × 10 s, 4 × 30 s, 14 × 240 s)	Correction: decay, attenuation, scatter and dead time	Manual Fixed-sized VOIs Based on anatomical locations
Kobayashi (2013) [23]	Osteoarthritis (hip)	SET-2400W instrument (Shimadzu, Kyoto, Japan)	PET	Dose: 185 MBq Scan time: static sweep 40 min after injection	Matrix: 128 × 128 Algorithm: OSEM Correction: attenuation	NS
Kobayashi (2015) [25]	Osteoarthritis (hip)	SET 2400 W instrument (Shimadzu, Kyoto, Japan)	PET	Dose: 185 MBq Scan time: static sweep 40 min after injection	Matrix: 128 × 128 Algorithm: OSEM	NS
Kobayashi (2015) [30]	Osteoarthritis (hip)	SET-2400W instrument (Shimadzu, Kyoto, Japan)	PET	Dose: 185 MBq Scan time: static sweep 40 min after injection	Matrix: 128 × 128 Algorithm: OSEM Correction: attenuation	Manual
Tibrewala (2019) [96]	Osteoarthritis (hip)	SIGNA 3 T time-of-flight (TOF) PET/MR (GE Healthcare, Milwaukee, WI)	PET/MRI	Dose: 247.97 ± 19.82 MBq Scan time: 45 min dynamic sequence (12 × 10 s, 6 × 30 s, 10 × 240 s)	NS	NS
Yellanki (2018) [25, 26]	Osteoarthritis (hip)	NS (part of CAMONA study)	PET/CT	Dose: NS Scan time: static sweep 90 min after injection	Corrections: attenuation, scatter, scanner dead time, and random coincidences	Manual and semi-automatic On CT images Based on a threshold of 150 HU and anatomical boundaries
Jena (2022) [24]	Osteoarthritis (knee)	Siemens ET/MRI system, Biograph mMR (Siemens, Erlangen, Germany)	PET/MRI	Dose: 185–370 MBq Scan time: static sweep 45 min after injection	Algorithm: OSEM with 3 iterations and 21 subsets, Gaussian smoothing	Manual On MRI images Based on anatomical locations
Jena (2023) [29]	Osteoarthritis (knee)	Siemens ET/MRI system, Biograph mMR (Siemens, Erlangen, Germany)	PET/MRI	Dose: 185–370 MBq Scan time: static sweep 45 min after injection	Algorithm: OSEM with 3 iterations and 21 subsets, Gaussian smoothing	Manual On MRI images Based on anatomical locations
Mackay (2021) [97]	Osteoarthritis (knee)	3 T PET-MRI platform (GE Signa PET-MR, GE Healthcare, Waukesha, WI)	PET/MRI	Dose: 90 MBq Scan time: 50 min dynamic sequence	Algorithm: TOF	Manual On MRI images
Savic (2016) [28]	Osteoarthritis (knee)	3 T PET-MR scanner (GE Healthcare, Milwaukee, Wisconsin)	PET/MRI	Dose: 340.4 MBq Scan time: 60 dynamic sequence (12 × 10,s 4 × 30 s, 14 × 240 s)	Algorithm: OSEM, TOF	Manual On MRI images Image-derived input function obtained from A. poplitea
Tibrewala (2020) [27]	Osteoarthritis (knee)	SIGNA 3 T time-of-flight (TOF) PET-MRI (GE Healthcare, Milwaukee, WI)	PET/MRI	Dose: 294.87 ± 59.78 MBq Scan time: dynamic sequence for 60 min after injection	Algorithm: OSEM 4 iterations and 28 subsets, TOF	NS
Watkins (2021) [98]	Osteoarthritis (knee)	3 T whole-body time-of-flight hybrid PET/MRI (GE Healthcare, Milwaukee, WI)	PET/MRI	Dose: 93 ± 4.4 MBq Scan time: 50 min dynamic sequence (20 × 1 s, 10 × 10 s, 10 × 30 s, 5 × 1 min 1 × 2 min)	Algorithm: TOF	Manual On MRI images According to existing radiological score subdivisions Image-derived input function obtained from A. poplitea
Watkins (2022) [99]	Osteoarthritis (knee)	3 T PET-MRI system (GE Healthcare, Milwaukee, WI)	PET/MRI	Dose: 89 ± 7.0 MBq Scan time: 50 min (20 × 1 s, 10 × 10 s, 10 × 30 s, 5 × 1 min 1 × 2 min)	Corrections: attenuation	Manual On MRI images According to grid zones based on anatomy
Christersson (2018) [58]	Osteomyelitis	GE Discovery ST16 hybrid (General Electric Medical Systems, Waukesha, WI, USA)	PET/CT	Dose: 2 MBq/kg Scan time: static sweep 60 min after injection	NS	NS
Freesmeyer (2014) [56]	Osteomyelitis	Biograph mCT 40 (Siemens Healthineers, Erlangen, Germany)	PET/CT	Dose: 200 MBq Scan time: Dynamic sequence of 5 min was performed after injection. Static sweep was then performed 30–45 min post-injection	Matrix: 200 × 200	Manual On CT images Based on anatomical boundaries and on CT affected areas
Reinert (2022) [57]	Osteomyelitis (jaw)	Biograph mCT (Siemens Healthineers, Erlangen, Germany)	PET/CT	Dose: 4 MBq/kg (284 ± 137 MBq) Scan time: static sweep 60 min after injection	NS	Semi-automatic On PET images Based on observed PET activity
Kubota (2015) [64]	Osteonecrosis (femoral head)	SET-2400W (Shimadzu, Kyoto, Japan)	PET	Dose: 185 MBq Scan time: static sweep 40 min after injection	Matrix: 128 × 128 Algorithm: OSEM Corrections: attenuation	NS
Aratake (2009) [65]	Osteonecrosis (knee)	SET-2400W (Shimadzu, Kyoto, Japan)	PET	Dose: 185 MBq Scan time: static sweep 40 min after injection	NS	NS
Schiepers (1998) [66]	Osteonecrosis (femoral head)	ECAT-931 PET (Siemens/CTI, Knoxville, Tennessee, USA)	PET	Dose: 300 – 370 MBq Scan time: static sweep 60 min after injection	NS	NS
Frost (2008) [71]	Osteoporosis	ECAT-951R PET (Siemens/CTI, Knoxville, Tennessee, USA)	PET	Dose: 90 MBq Scan time: 60 min dynamic sequence (12 × 10 s, 4 × 30 s, 14 × 240 s)	Corrections: attenuation	Semi-automatic toll based on a threshold of 50% of the maximum bone activity in each image set Image-derived input function from the aorta abdominalis corrected using venous blood samples
Jassel (2019) [67]	Osteoporosis	GE Discovery (General Electric Medical Systems, Waukesha, WI, USA)	PET/CT	Dose: 90 MBq for the lumbar spine scan and 180 MBq for the hip scan Scan time: dynamic scan for 60 min after injection	Algorithm: Back-projection using a 6.3-mm Hanning filter Corrections: Scattered radiation and attenuation	Manual On CT images Based on anatomical boundaries
Park (2023) [100]	Osteoporosis	GE Discovery (GE Healthcare, Chicago, Illinois, USA)	PET/CT	Dose: 2.2 MBq/kg Scan time: static sweep 90 min after injection	NS	Manual On CT images Based on anatomical boundaries
Puri (2021) [101]	Osteoporosis	GE Discovery PET-CT scanner (General Electric Medical Systems, Waukesha, WI, USA)	PET/CT	Dose: 180 MBq Scan time: 60 min dynamic sequence (24 × 5 s, 4 × 30 s, 14 × 240 s)	Corrections: decay, attenuation	Manual On CT images Based on anatomical boundaries Arterial input function was estimated using a semi-population method
Rhodes (2020) [102]	Osteoporosis	GE Discovery STE, VCT, RX, and 690/710 (General Electric Medical Systems, Waukesha, WI, USA)	PET/CT	Dose: 2.2 MBq/kg Scan time: static sweep 90 min after injection	NS	Manual On CT images Based on anatomical boundaries with 100 HU threshold for whole bone and 300 HU for cortical bone
Uchida (2009) [103]	Osteoporosis (Alendronate treatment)	Advance system (GE Healthcare)	PET	Dose: 185 MBq Scan time: static sweep 50 min after injection	Algorithm: iterative, 14 subsets and 2 iterations	Manual with fixed-sized VOI On PET images
Frost (2003) [104]	Osteoporosis (Risendronate treatment)	ECAT-951R PET (Siemens/CTI, Knoxville, Tennessee, USA)	PET	Dose: 90 MBq Scan time: 60 min dynamic sequence (12 × 10 s, 4 × 30 s, 14 × 240 s)	Algorithm: back-projection with Hann filter Corrections: attenuation	Semi-automatic tool based on a threshold of 50% of the maximum bone activity in each image set Image-derived input function from the aorta abdominalis corrected using venous blood samples
Frost (2011) [68]	Osteoporosis (Teriparatide treatment)	GE Discovery PET/CT (General Electric Medical Systems, Waukesha, WI, USA)	PET/CT	Dose: 90 MBq Scan time: 60 min dynamic sequence (24 × 5 s, 4 × 30 s, 14 × 24 s) followed by a static scan of the femur and pelvis	Corrections: attenuation	Manual On CT images Based on anatomical boundaries Input function was estimated using a semi-population curve method
Siddique (2011) [105]	Osteoporosis (Teriparatide treatment)	GE Discovery PET/CT scanner (General Electric Medical Systems, Waukesha, Wisconsin, USA)	PET/CT	Dose: 90 MBq Scan time: 60 min dynamic sequence (24 × 5 s, 4 × 30 s, 14 × 240 s)	NS	Manual On PET/CT images Based on anatomical boundaries Arterial plasma input function was calculated using a semi-population method, corrected with venous samples
Fushimi (2022) [106]	Osteoporosis (Zolendronic acid & denosumab treatment)	Biograph mCT 64-slice PET/CT (Siemens Medical Solutions USA, Knoxville, TN)	PET/CT	Dose: 5 MBq/kg Scan time: static sweep 60 min after injection	Algorithm: TOF	Manual Based on anatomical boundaries
Puri (2012) [69]	Osteoporosis (postmenopausal bone turnover)	GE Discovery ST scanner (General Electric medical Systems, Waukesha, WI, USA)	PET/CT	Dose: 90 MBq for lower spine scan and 180 MBq for the hip scan Scan time: 60 min dynamic sequence (24 × 5 s, 4 × 30 s and 14 × 240 s)	Mode: 2-dimensional Corrections: scatter, attenuation	Manual On CT images Based on anatomical boundaries
Cook (2000) [21]	Osteoporosis (postmenopausal bone turnover)	ECAT-951R PET (Siemens/CTI, Knoxville, Tennessee, USA)	PET	Dose: 180 MBq Scan time: 60 min dynamic sequence (24 × 5 s, 4 × 30 s and 14 × 240 s)	Correction: attenuation	Manual Arterial input function was based on a mean population input function corrected for plasma samples obtained at 30, 40 and 50 min
Frost (2004) [107]	Osteoporosis (postmenopausal bone turnover)	ECAT-951R PET (Siemens/CTI, Knoxville, Tennessee, USA)	PET	Dose: 90 MBq Scan time: 60 min dynamic sequence (12 × 10 s, 4 × 30 s, 14 × 240 s)	Correction: attenuation	Semi-automatic tool based on a threshold of 50% of the maximum bone activity in each image set Image-derived input function obtained from the abdominal aorta and corrected using venous blood samples
Frost (2009) [108]	Osteoporosis (postmenopausal bone turnover)	ECAT-951R PET (Siemens/CTI, Knoxville, Tennessee, USA)	PET	Dose: 90 MBq Scan time: 60 min dynamic sequence (12 × 10 s, 4 × 30 s, 14 × 240 s)	Correction: attenuation	Semi-automatic tool based on a threshold of 50% of the maximum bone activity in each image set Image-derived input function obtained from the abdominal aorta and corrected using venous blood samples
Puri (2013) [70]	Osteoporosis (postmenopausal bone turnover)	Discovery ST (General Electric Medical Systems, Waukesha, WI, USA)	PET/CT	Dose: 90 MBq for the lumbar spine scan and 180 MBq for the hip scan Scan time: dynamic sequence for 60 min after injection	Matrix: 128 × 128 Mode: 2-dimensional Corrections: attenuation	Semi-automatic On CT images Based on anatomical boundaries
Cook (2002) [109]	Paget’s disease	ECAT-951R PET scanner (Siemens/CTI Inc., Knoxville, TN, USA)	PET	Dose: 180 MBq Scan time: 60 min dynamic sequence (12 × 10 s, 4 × 30 s,14 × 240 s)	Correction: attenuation	Manual On CT images Image-derived input function from the aorta
Installe (2015)	Paget’s diseases	ECAT 961 PET scanner (Siemens/CTI Inc., Knoxville, TN, USA)	PET	Dose: 397 ± 40.7 MBq Scan time: 60 min dynamic sequence (12 × 10 s, 4 × 30 s,14 × 240 s)	Correction: dead time, random coincidences, scatter, decay, attenuation	Manual On PET images
Waterval (2013) [35]	Otosclerosis	Gemini TF (Philips, Best, The Netherlands)	PET/CT	Dose: 200 MBq Scan time: 60 min after injection	Algorithm: OSEM Corrections: random events, scattered radiation and attenuation	Manual On CT images Based on anatomical boundaries
Draper (2012) [36]	Patellofemoral pain	GE Discovery LS (GE Healthcare, Milwaukee, WI)	PET/CT	Dose: 185–370 mBq (2.96 mBq/kg) Scan time: 69 ± 23 min after injection	Matrix: 128 × 128 Time/bed position: 5 min acquisition Algorithm: OSEM	Manual On CT images Based on anatomical boundaries
Graf (2020) [74]	Primary hyperparathyroidism and brown tumors	Discovery VCT, Discovery STE (GE Healthcare, Waukesha, WI)	PET/CT	Dose: 100 MBq Scan time: 30 min after injection	Matrix: 256 × 256 Algorithm: OSEM	Manual Unclear on which images Based on tumor locations
Hochreiter 2019 [110]	Reverse shoulder arthroplasty	Discovery 710 (GE Healthcare, Milwaukee, WN)	PET/CT	Dose: 150 MBq Scan time: static sweep 60 min after injection	Algorithm: OSEM	Manual On CT images Based on a 50% threshold of the PET activity in the area of interest
Jonnakuti (2018) [111]	Rheumatoid arthritis (knee arthrosis)	NS	NS	NS	NS	Manual On CT images Based on a threshold of 150 HU and on anatomical boundaries
Park (2021) [33]	Rheumatoid arthritis	Biograph mCT 20 (Siemens Healthineers, Knoxville, TN, USA)	PET/CT	Dose: 185 MBq Scan time: static sweep 57 ± 5 min after injection	Algorithm: Iterative reconstruction algorithm Corrections: attenuation	Manual On CT images Based on anatomical boundaries
Reddy (2023) [112]	Rheumatoid arthritis	Biograph 64 Hybrid PET/CT Imaging System (Siemens Medical Solutions, Inc. Malvern, PA, USA)	PET/CT	Dose: 2.96 MBq/kg Scan time: static sweep 90 min after injection	Corrections: scatter, random coincidences, dead time, attenuation	Manual On CT image Based on anatomical boundaries and thresholds of 150 HU up to 1500 HU
Watanabe (2016) [34]	Rheumatoid arthritis	SET 2400 W (Shimadzu, Kyoto, Japan)	PET/CT	Dose: 185 MBq Scan time: static sweep 40 min after injection	Corrections: attenuation	Manual On PET images Based on observed PET activity and anatomical boundaries
Aaltonen (2020) [14]	Renal osteodystrophy	Discovery VCT scanner (GE Healthcare)	PET/CT	Dose: 200 MBq Scan time: 60 min dynamic sequence (24 × 5 s, 4 × 30 s, 14 × 240 s)	Corrections: attenuation	Manual On CT images Based on anatomical boundaries Image-derived input function from the abdominal aorta
Aaltonen (2021) [13]	Renal osteodystrophy	Discovery VCT scanner (GE Healthcare)	PET/CT	Dose: 200 MBq Scan time: 60 min dynamic sequence (24 × 5 s, 4 × 30 s, 14 × 240 s)	Corrections: attenuation	Manual On CT images Based on anatomical boundaries Image-derived input function from the abdominal aorta
Frost (2013) [17]	Renal osteodystrophy	GE Discovery PET/CT scanner (General Electric Medical Systems, Waukesha, WI)	PET/CT	Dose: 90 MBq Scan time: 60 min dynamic sequence (12 × 10 s, 4 × 30 s, 14 × 240 s)	Corrections: attenuation	Semi-automatic tool based on a threshold of 50% of the maximum bone activity in each image set Image-derived input function obtained from the abdominal aorta and corrected using venous blood samples
Fuglo (2023) [113]	Renal osteodystrophy	Siemens Biograph mCT (Siemens Healthineers, Erlangen, Germany)	PET/CT	Dose: 200 MBq Scan time: 60 min dynamic sequence (4 × 30 s, 8 × 60 s, 12 × 240 s)	Matrix: 400 × 300 Algorithm: OSEM, 4 iterations and 21 subsets	Manual Based on anatomical boundaries Imaged derived input function from the A. Iliaca
Vrist (2021) [15]	Renal osteodystrophy	Siemens Biograph mCT-4R 64 slice PET/CT (Siemens Healthineers, Erlangen, Germany)	PET/CT	Dose: 150 MBq Scan time: 60 min dynamic sequence (203x, 12 × 5 s, 4 × 30 s, 14 × 240 s)	Correction: attenuation	Manual On PET/CT images Based on anatomical boundaries Image-derived input function obtained from the left ventricle and corrected using venous blood samples
Vrist (2022) [16]	Renal osteodystrophy	Siemens Biograph mCT-4R 64 slice PET/CT (Siemens Healthineers, Erlangen, Germany)	PET/CT	Dose: 150 MBq Scan time: 60 min dynamic sequence (203x, 12 × 5 s, 4 × 30 s, 14 × 240 s)	Correction: attenuation	Manual On PET/CT images Based on anatomical boundaries Image-derived input function obtained from the left ventricle and corrected using venous blood samples
Constantinescu (2020) [50]	Spinal interbody fusion (posterior lumbar interbody fusion)	Discovery LS 690/710 (GE Healthcare, Chicago, IL)	PET/CT	Dose: 2.2 MBq/kg Scan time: static sweep 90 min after injection	Corrections: attenuation, scatter, random coincidences, and scanner dead time	Manual and semi-automatic On CT images Based on anatomical boundaries
Peters (2015) [51]	Spinal interbody fusion (posterior lumbar interbody fusion)	Gemini TF PET/CT (Philips, The Netherlands)	PET/CT	Dose: 156–263 MBq Scan time: static sweep 60 min after injection	Algorithm: TOF Corrections: attenuation	Manual On CT images
Peters (2015) [52]	Spinal interbody fusion (posterior lumbar interbody fusion)	Gemini TF PET/CT (Philips, The Netherlands)	PET/CT	Dose: 156–214 MBq Scan time: 30 min dynamic sequence (6 × 5 s, 3 × 10 s, 9 × 60 s, 10 × 120 s)	Algorithm: blob-os-TF	Manual On CT images Image-derived input function obtained from the abdominal aorta
El Yaagoubi (2022) [49]	Spinal fusion (aseptic pseudoarthrosis)	Discovery IQ (Discovery IQ; GE Healthcare, Milwaukee, WN)	PET/CT	Dose: 2.2 MBq/kg Scan time: static sweep 60 min after injection	NS	Manual On PET images Based on observed PET activity
Lee (2019) [55]	Surgical-site infection	Biograph mCT 128 (Siemens Healthcare, Knoxville, TN)	PET/CT	Dose: 185 MBq Scan time: Early-phase imaging was performed after injection (sequence times not reported). Static sweep was performed approximately 45 min post-injection	Algorithm: Iterative algorithm using point-spread-function modeling and time-of-flight reconstruction Corrections: attenuation	Manual On CT images Based on anatomical boundaries
Lee (2013) [63]	Temporomandibular joint disorder	Biograph™ 40 (Siemens Medical Solutions, Hoffman Estates, IL)	PET/CT	Dose: 185–370 MBq Scan time: static sweep 40 min after injection	Matrix: 128 × 128 Algorithm: OSEM Correction: attenuation	Manual On CT images Base on anatomical locations
Suh (2018) [62]	Temporomandibular joint disorder	Discovery (GE Healthcare, Milwaukee, WI, USA)	PET/CT	Dose: 5.18 MBq/kg Scan time: 60 min after injection	Matrix: 128 × 128 Mode: 3-dimensional Algorithm: ordered-subset iteration algorithm Corrections: attenuation and scatter	Manual On CT images Based on anatomical boundaries
Lundblad (2017) [47]	Tibia bone healing (complex fractures, osteomyelitis, osteotomies)	Biograph 64 Truepoint TrueV (Siemens Medical Solutions, Erlangen, Germany)	PET/CT	Dose: NS Scan time: Dynamic sequence of 45 min after injection, followed by static sweep at 60 min	Algorithm: “suitable reconstruction algorithm was determined via phantom studies” Corrections: attenuation	Manual On CT images Based on anatomical boundaries
Lundblad (2015) [114]	Tibia bone healing (with Taylor Spatial Frame)	Biograph 64 Truepoint TrueV (Siemens Medical Solutions, Erlangen, Germany) Discovery 710 and Discovery MI DR (GE Healthcare, Waukesha, WI, USA)	PET/CT	Dose: 2 MBq/kg Scan time: Dynamic sequence of 45 min after injection, followed by static sweep after 60 min	Algorithm: suitable reconstruction algorithm was determined via phantom studies Corrections: attenuation	Manual On CT images Based on anatomical boundaries
Sanchez-Crespo (2017) [48]	Tibia bone healing (with Taylor spatial frame)	Biograph 64 TruePoint True V (Siemens Medical Solutions, Erlangen, Germany)	PET/CT	Dose: 2 MBq/kg Scan time: 60 min dynamic sequence (6 × 10 s, 4 × 30 s, 7 × 60 s, 5 × 180 s and 4 × 300 s)	Matrix: 168 × 168 Mode: 2-dimensional Algorithm: OSEM, 4 iterations 8 subsets Corrections: attenuation, decay, random coincidences	NS
Rauscher (2015) [115]	Unclear foot pain	Biograph mCT (Siemens Healthineers, Knoxville, TN, USA)	PET/CT	Dose: 133 ± 68 MBq Scan time: 75 ± 18 min after injection	Mode: 3-dimensional Algorithm: OSEM Corrections: attenuation via attenuation maps by bilinear transformation	Semi-automatic On PET images An automatic isocontour based on 50% of the SUV_max_ manually placed
Lima (2018) [61]	Unilateral condylar hyperplasia	Discovery 710 TF (GE Medical System, Waukesha, WI)	PET/CT	Dose: 5.3 MBq/Kg (group A) or 2.9 MBq/Kg (group B) Scan time: 60 min after injection of 5.3 MBq/Kg	Algorithm: iterative reconstruction algorithm, time-of-flight and point-spread-function recovery resolution information	Manual On CT images Based on anatomical boundaries
Ahmed (2016) [60]	Unilateral condylar hyperplasia	Discovery (GE, Schenectady, New York, USA)	PET/CT	Dose: 370 MBq Scan time: 45 min after injection	Algorithm: OSEM Mode: 3-dimensional Corrections: attenuation	Manual On PET images Based on observed PET activity
Saridin (2009) [59]	Unilateral condylar hyperplasia	ECAT EXACT HR + scanner (Siemens/CTI, Knoxville, TN)	PET/CT	Dose: 100 MBq Scan time: 60 min dynamic sequence (6 × 5 s, 6 × 10 s, 3 × 20 s, 5 × 30 s, 5 × 60 s, 8 × 150 s, and 6 × 300 s)	Matrix: 128 × 128 Algorithm: OSEM, 2 iterations, 16 subsets	Manual Placement of fixed-sized VOI around anatomical locations
Schiepers (1997) [6]	Various bone disorders	ECAT-931 PET system (Siemens/CTI, Knoxville, Tennessee, USA)	PET	Dose: 300 – 370 MBq Scan time: static sweep 60 min after injection	NS	NS

### Quality assessment

Most articles (90/92) were cross-sectional or cohort studies and were assessed using the National Heart, Lung, and Blood Institute (NHLBI) Study Assessment Tool [12]. The remaining two articles were considered to describe a randomized control trial (RCT) and were, therefore, assessed using NHLBI’s RCT study assessment tool. 83 studies were of good quality (90.2%), 9 of fair quality (9.8%) and none were of poor quality. The rating for each study can be found in Supplementary Material S2. No articles were excluded based on their quality rating.

### Purpose of [^18^F]NaF uptake quantification

**Fig. 3:**
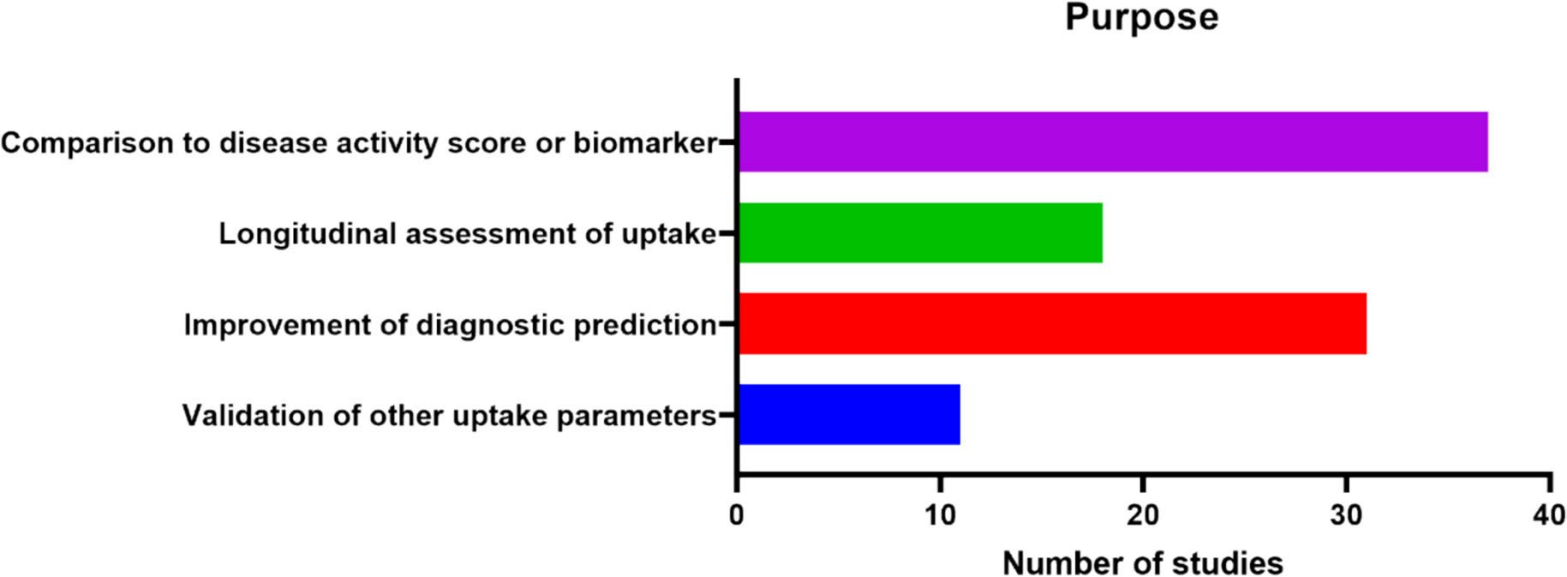
Overview of categories of quantitative [^18^F]NaF PET studies included. The largest group of studies compared uptake with disease activity or a different biomarker. Other purposes were to improve diagnosis, to assess bone metabolism over time (longitudinal assessment), and to validate simplified parameters

Evaluation of [^18^F]NaF uptake as a measure of bone metabolism is of interest in various bone-related disorders. A full overview of the studies included and what they investigated through quantification of [^18^F]NaF uptake can be found in Table 1. Studies performing [^18^F]NaF uptake measurements can roughly be divided in four categories: cross-sectional analysis correlating uptake parameters with existing biomarkers or disease activity scores, longitudinal analysis with multiple [^18^F]NaF PETs to evaluate disease activity over time, incorporation of uptake parameters to improve the diagnostic procedure, and comparison of more simplified quantitative parameters to the quantification of uptake by full kinetic analysis with NLR (Fig. 3).

#### Comparison with existing biomarkers or diseases activity scores

The gold standard for measurement of bone turnover is histological analysis of bone obtained through biopsies. Comparison of bone turnover as measured by [^18^F]NaF uptake on the PET scan with histological analysis of bone was only performed in patients with renal osteodystrophy. NLR-derived *K*_*i*_ and Patlak-derived *K*_*i*_ correlated significantly with the histological classification of renal osteodystrophy as well as specific histological markers of bone turnover such as bone formation rate, activation frequency, mineralized surface, and mineral acquisition apposition rate [13–17]. NLR-derived *K*_*i*_ and Patlak-derived *K*_*i*_ also correlated with other markers of turnover in renal dystrophy such as parathyroid hormone (PTH) and alkaline phosphatase (ALP) [13].

In metabolic bone diseases where histological analysis was not performed, frequently serum markers were used to evaluate disease activity. For metabolic bone diseases that manifest themselves throughout the body, bone turnover markers such ALP and procollagen type 1 propeptide (P1NP) are frequently used in clinical practice. In fibrous dysplasia (FD), for example, ALP and P1NP correlated with the total metabolic active volume (MAV); defined as the volume surpassing a pre-set SUV threshold [18]. To better encapsulate overall disease activity, the total lesion fluorination (TLF), defined as MAV multiplied by SUV_mean_, was also examined. TLF proved to be the most reproducible with high inter-observer agreement for FD burden, while also reflecting changes in serum biomarkers [19]. High MAV and TLF were also correlated with FD related complications such as fracture risk and frequency of surgical interventions [20]. In studies examining Paget’s disease, NLR-derived *K*_*i*_ in pagetic bone was significantly higher than that in non-pagetic bone and correlated with serum ALP levels [21, 22].

Alternatively, [^18^F]NaF uptake measures have been correlated with an existing disease activity score or grade. This was done most extensively in osteoarthritis, with various measures of [^18^F]NaF uptake being compared with various markers of disease. For instance, in hip osteoarthritis, SUV_max_ correlated with the Kellgren–Lawrence grade, a semi-quantitative radiographic osteoarthritis score [23, 24]. Increasing SUV_max_ and SUV_mean_ were correlated with increasing pain scores [23, 25] and increased BMI [26]. Similarly, for osteoarthritis in the knee, increased SUV_max_, SUV_mean_ correlated with an increased MRI osteoarthritis knee score (MOAKS) and was associated with cartilage and osteophytic lesions [27–29]. Inverting this relationship showed that a SUV_max_ > 6.5 was indicative of the presence of osteoarthritis in the joint [30].

Disease activity scores have also frequently been used in rheumatic diseases. The bath ankylosing spondylitis disease activity index (BASDAI) score was correlated with various approaches attempting to discern [^18^F]NaF uptake in ankylosing spondylitis (AS) studies. Lee et al. calculated the lesion-to-bone ratio by dividing the SUV_max_ of the lesion with the SUV_mean_ of the L5 vertebra, focusing on the posterior vertebral joints, though no correlation with the BASDAI score was found [31]. Kim et al. measured the *K*_*i*_ with NLR analysis as well as the SUV_mean_ in the spine, finding a positive correlation between both parameters and the BASDAI score [32]. Similarly, a rheumatoid arthritis disease score, the DAS28-ER, correlated positively with the summed SUV_max_ of the 28 joints included in the score [33, 34].

Other studies have evaluated fluoride uptake against a marker or scale associated with a disease-specific condition. Increased SUV_mean_ in the otic capsule was graded against increased hearing loss in otosclerosis patients [35]. In patients with patellofemoral pain, increased SUV_mean_ and SUV_peak_ in the patellofemoral joint correlated with more pain as measured by standardized pain questionnaires [36]. Finally, after ankle trauma, increased SUV_max_, SUV_mean_, and TLA correlated with a decrease in range of motion [37].

#### Longitudinal assessment

Performing multiple PET/CT scans over time provides a means to study the pathophysiology of diseases affecting bone turnover and make it possible to evaluate disease progression, treatment response, and healing. Bone healing over time has been investigated in various surgery-related studies. Tracer uptake measured in bone grafts decreased over time, with 25% decrease 6 months after surgery and a 60–65% decrease 2 years after surgery. The measured decrease was comparable between the various reported uptake parameters with high correlation between themselves [38]. Multiple studies were performed with [^18^F]NaF PET following a hip arthroplasty, inferring an elevated NLR-derived *K*_*i*_, SUV_max_ or SUV_mean_ to be an indication of successful bone healing [39–42]. Subsequently, SUV_max_ was used to compare the effectiveness of different types of cementation and implants in hip arthroplasties [43, 44]. However, an increased SUV_max_ was also found to be predictive of sceptic loosening of the prosthesis [45, 46].

Lundblad et al. studied tibial bone healing with a Taylor spatial frame using PET/CT, finding a SUV_max_ difference > 0.18 per day to be indicative of faster bone healing. However, there was no consistent pattern throughout the bone-healing process, nor an association with a specific treatment outcome [47, 48]. The post-operative course after posterior lumbar interbody fusion has also been investigated. An increased SUV_max_ at the endplates was associated with subsidence of the endplates on CT [49, 50]. A higher SUV_max_ was also found around the cage in the intervertebral disc space in patients requiring revision surgery [51, 52].

In fibrodysplasia ossificans progressiva, a PET/CT study over time elucidated the pathophysiology further by demonstrating progression in the absence of any clinical signs, but also showing that heterotopic [^18^F]NaF uptake as measured by a SUV_peak_ > 8.2 was predictive of heterotopic ossification [53]. One step further, [^18^F]NaF uptake has also been used to evaluate treatment response in other metabolic bone diseases. In ankylosing spondylitis, PET/CT was used to evaluate treatment response to anti-TNF treatment. [^18^F]NaF uptake in costovertebral and SI joints decreased significantly in clinical responders in contrast to non-responders [54].

#### The diagnostic procedure

By comparing uptake in disease-affected tissue areas with that in healthy tissue areas, multiple studies used [^18^F]NaF PET/CT to try and improve the diagnostic procedure. In osteomyelitis studies, [^18^F]NaF PET/CT has been used to identify infected areas by measuring SUV_max_ and SUV_mean_, and comparing it with uptake in healthy bone tissue [55–57]. [^18^F]FDG PET/CT can already be used to identify areas of infection in osteomyelitis, but with low specificity as it detects all metabolically active cells. As such, Christersson et al. combined the [^18^F]FDG PET/CT with [^18^F]NaF PET/CT, finding a positive correlation between [^18^F]NaF and [^18^F]FDG PET/CT SUV_max_ and SUV_mean_ in affected areas, but also improving outcome of the surgical procedures by better identification of the area requiring resection [58].

In unilateral condylar hyperplasia, uptake measured with NLR-derived *K*_*i*_ [59] and SUV_max_ [60, 61] were compared between the affected and unaffected (contralateral) condyles within the same patient, finding an increased uptake in the affected condyle and concluding that a difference of > 10% was a mark of active disease. Similarly, in temporomandibular joint (TMJ) disorders, SUV_max_ was significantly higher in TMJ osteoarthritis than in asymptomatic TMJ [62] with an elevated TMJ-to-skull ratio (SUV_mean_ of the TMJ divided by the SUV_mean_ of a separate are in the mandibular) indicating the presence of TMD osteoarthritis with a sensitivity of 89% and a specificity of 81% [63]. Conversely, a decreased NLR-derived K_i_ and SUV_max_ was indicative of osteonecrosis, also correlating with the progression according to the Ficat classification stages [64–66].

A different approach to better detect disease was by generating cut-off values, sometimes followed by ROC analysis. In osteoarthritis, a SUV_max_ > 6.5 was found to be indicative of osteoarthritis in the joint [30] with a SUV_max_ > 7.2 being predictive of worsening pain and minimal joint space narrowing [25]. Similarly in hip arthroplasty studies, a SUV_max_ > 4.4–5.0 was found to be indicative of sceptic loosening of the prosthesis [45, 46].

#### Comparison with full kinetic analysis

Several studies compared the performance of more simplified uptake parameters with NLR-derived *K*_*i*_ to validate their potential use as a surrogate marker. Twenty-nine studies performed full kinetic analysis measuring NLR-derived *K*_*i*_ in twenty-six different conditions. Eleven of these studies compared various simplified fluoride uptake parameters with the gold standard NLR-derived K_i_.

There was also variety in the use of input curves for calculating the *K*_*i*_. Arterial input curves can be obtained through continuous arterial sampling through an arterial line, though as this is rather invasive, other alternative methods have been developed to estimate the arterial curves. Some studies use venous samples to correct arterial curves obtained from separate population-based studies to estimate arterial input curves [17, 67, 68]. Other studies used an image-derived arterial input curve, sometimes corrected with venous samples, to serve as an acceptable alternative for invasive arterial sampling [69].

Vrist et al. found a high correlation between Patlak-derived *K*_*i*_ and NLR-derived *K*_*i*_ in patients with renal osteodystrophy [15]. Brenner et al. also found a high correlation between SUV_mean,_ Patlak-derived *K*_*i*_ and NLR-derived *K*_*i*_ when evaluating bone graft healing after surgery, validating SUV_mean_ as a valid parameter to track bone graft healing in future studies. In cross-sectional analyses, Puri et al. found high correlations between NLR-derived *K*_*i*_ and both Patlak-derived *K*_*i*_ and SUV_mean_ when evaluating postmenopausal bone turnover in 12 patients [70]. Also in osteoporosis, Frost et al. found a good correlation between NLR-derived *K*_*i*_ and both Patlak-derived *K*_*i*_ and SUV_mean_ [71] with a positive correlation between NLR-derived *K*_*i*_ and BMD being established later [67]. However, when evaluating the response to teriparatide treatment in osteoporosis, they found a poor correlation between SUV_mean_ and NLR-derived *K*_*i*_ [68], demonstrating the necessity to reevaluate the suitability of simplified markers when using them as a marker for treatment response. A nice example of this is a study in AS, where Bruijnen et al. first validated the use of SUV^AUC^ (area under the curve) as a measure of [^18^F]NaF uptake by comparison with NLR-derived *K*_*i*_ over time, before using SUV^AUC^ to evaluate the response of patients with AS to anti-TNF treatment [54].

### PET details

Table 2 summarizes the more technical (protocol) details. Of the 92 studies included, 56 used PET/CT systems to study the bone disease of interest, 23 used PET only systems, and 11 used PET/MRI systems. Scanner type and model were reported in most of the studies included (90/92). This was also true for the dose of [^18^F]NaF injected (87/92) and for the interval between injection and scan (89/92). Thirty-six of these studies consisted of dynamic scans, where the PET acquisition started at the time of injection of the tracer. The injected dose reported ranged from 111 to 421 MBq, with most studies reporting an injected activity close to 185 MBq, in line with current EANM guidelines. The start time of the static sequence varied greatly from 30 up to 90 min post-tracer injection.

There was also a wide variety in approaches toward defining VOI. Broadly speaking, VOI methods consisted either of manual delineation on either CT or PET images, a semi-automated approach based on a pre-specified threshold of CT- or PET-related values such as HU or SUV, or a combination of these methods. The chosen HU thresholds varied from 80 up to 150 HU, but often were not reported at all. No MRI-based values were reported to discern between tissues and subsequently determine the VOI. Some studies based on their VOI clearly defined anatomical landmarks or according to pre-existing radiological classification scores. Not all studies provided sufficient details on definition and subsequent delineation of volumes of interest to be considered reproducible.

## Discussion

**Fig. 4:**
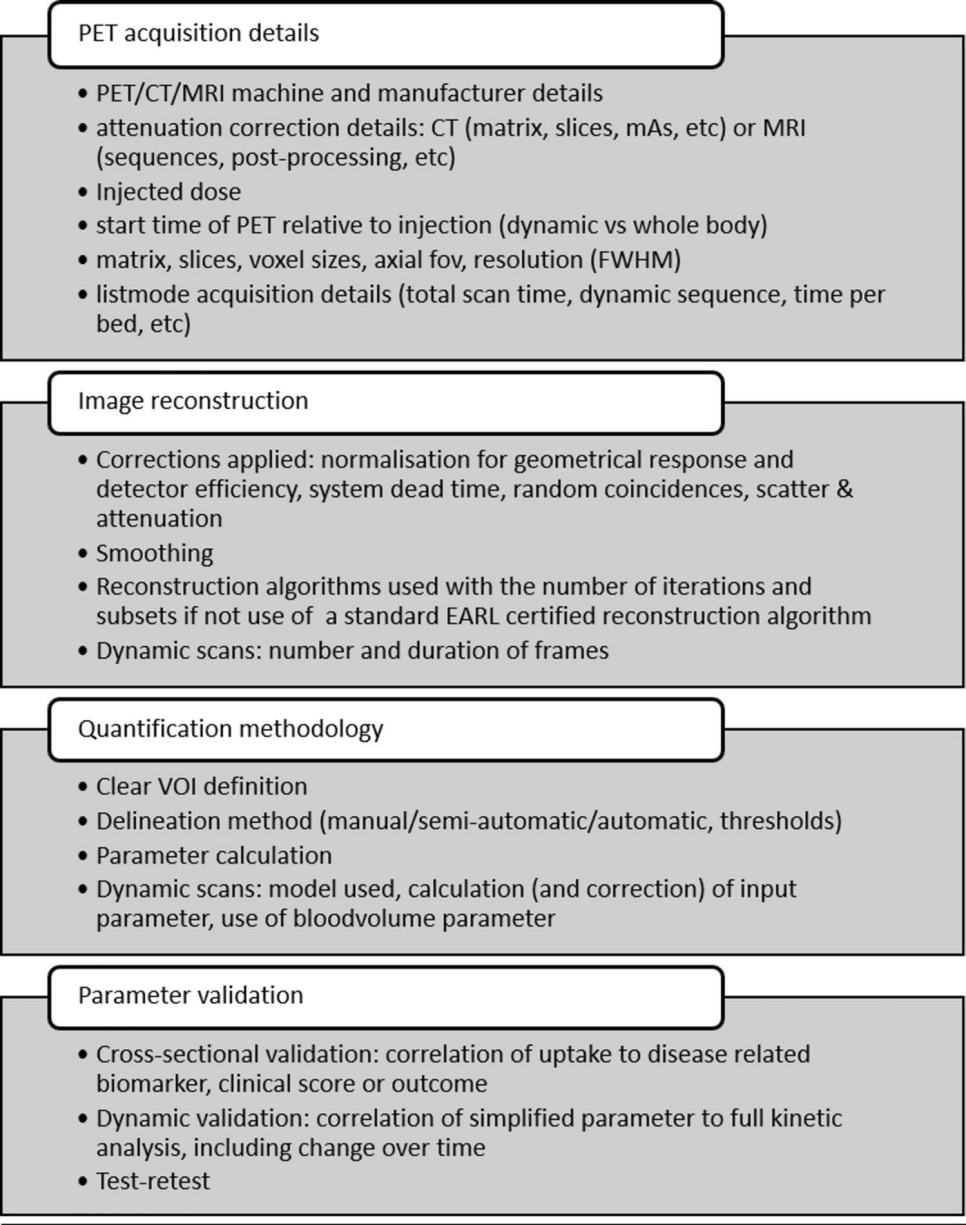
Information to be considered for documentation to reliably reproduce the quantification of an uptake parameter obtained with the [18F]NaF PET, PET/CT or PET/MRI

The aim of this systematic review was to investigate which (semi-)quantitative parameters were used to measure [^18^F]NaF uptake in bone disorders and for what purpose. The literature search identified 92 studies that examined 29 different bone disorders. Most of the studies used SUV to quantify [^18^F]NaF uptake, comparing its uptake with either a separate disease marker or outcome to establish clinical relevance. In addition to comparing uptake with a separate clinical marker, to properly establish whether a change in SUV is indeed due to a change in bone formation, studies using SUV (or other semi-quantitative parameters) should ideally validate it by comparison with NLR-derived K_i_ obtained, obtained from a dynamic analysis, to exclude effects of confounding factors affecting tracer distribution. Unfortunately, there was considerable variation in how detailed [^18^F]NaF PET/CT protocols and data analysis procedures were reported in the studies that were included. Figure 4 provides a list of details that should be reported to reliably interpret an [^18^F]NaF uptake parameter as study outcome. This list is by no means exhaustive, as which details are reported will depend on the type and goal of a study.

The [^18^F]NaF PET scan visualizes areas of bone formation and can, thus, be used to investigate pathophysiological processes in which this is disrupted. By quantifying the amount of tracer uptake, the rate of bone formation can be measured in a specific area, providing an advantage over regular bone turnover markers in serum, which are non-specific in that respect. Most of the studies in this review measured [^18^F]NaF uptake in a certain area of interest and correlated that with a more general disease marker, such as validated disease activity questionnaires completed by patients, blood serum markers associated with disease activity or other radiographical findings (37/92). Uptake in a certain area affected by the disease was also often compared with that in an area of healthy tissue, sometimes to establish what level of uptake is pathological with the aim to subsequently generate cut-off values predictive of the presence of a particular disease (26/92). In cross-sectional analyses, it is possible to correlate two markers at a single point, but by performing multiple [^18^F]NaF PET/CT scans over time in a longitudinal analysis, it is possible to determine whether a change in uptake also correlates with a change in other disease markers. Although quite a few studies evaluated the change in uptake over time (18/92) most of these studies used this to evaluate the post-operative healing process without necessarily comparing the measured uptake with any other marker or outcome.

Most studies included in this review used SUV to quantify tracer uptake. Several variations of SUV exist, with SUV_max_ being the most frequently reported, followed by SUV_mean_ and SUV_peak_. This is not surprising, as SUV measurements are simple and can be obtained without blood sampling. Moreover, SUV calculations can be performed in conjunction with a whole-body scan, making it easier to assess a larger area of the body. Simplified parameters obtained with a static scan such as SUV have several benefits over parameters obtained through full kinetic analysis and are, therefore, the preferred method for quantifying tracer uptake in routine clinical practice. In general, it is less burdensome on the patients undergoing the scan due to a shorter scanning time and no requirement for arterial or venous blood sampling. However, SUV is also prone to bias with factors affecting its accuracy. This may be due to patient-related factors affecting the biodistribution of [18F]NaF such as regional blood flow, “steal” phenomenon (systemic vasodilation leading to a transient decrease of perfusion in the area of interest) or systemic tracer clearance, but it can also be due to technical factors such as varying times between injection and scanning and injected dose. One approach is to validate a simplified parameter against an unrelated marker considered to be a relevant measure of disease activity. Many of the studies in our review used this approach, comparing uptake measured through SUV against various disease activity scores or relevant biomarkers [18, 23, 35, 54, 65, 72, 74].

A different approach to validate the use of a simplified parameter is by comparing its performance to NLR-derived K_i_ obtained through full kinetic modeling. In studies with different tracers, there have been examples of cases where the change in a simplified parameter poorly reflected the change in uptake as measured by full kinetic analysis, leading to the use of another simplified parameter [73, 75, 76]. Moreover, a new therapy may also affect biological factors affecting the biodistribution of the tracer. A change in blood flow may result into a change in the rate of tracer uptake, without in fact changing the total amount of tracer uptake. Full kinetic modeling can evaluate whether therapy-induced changes are due to a real therapy effect or due to confounding factors such as a change in perfusion. Ideally, the performance of both approaches can be assessed to justify the use of more simplified parameters before they are used as routine outcome measures. However, the choice of quantification method (and validation of it) depends on the application of the [^18^F]NaF PET. In the case of measuring if there is increased tracer uptake than normal in the area of interest, a static scan with SUV measurement is sufficient and preferable over extensive kinetic modeling analyses.

A key unexpected finding of this survey was the fact that the description of the methodology used was insufficient in many articles, making it impossible to reliably reproduce reported findings. Table 2 summarizes the details of the studies and shows a wide variety on what was reported. For any quantitative imaging study, the description of the methodology should be detailed enough so that the study can be reproduced. Two aspects require better reporting in most of the studies included. First, each study should clearly define the volume studied (VOI) and how it was delineated on the scans. This can be done in several different ways. Some studies used clear anatomical boundaries to define a VOI, others used a threshold based on a radiological unit such as HU or SUV. The description should be sufficiently detailed so that anyone can delineate a similar VOI if necessary. Second, the choice and calculation of the uptake parameter should be specified. For example, SUV can be corrected for body weight, lean body mass or body surface area. Studies reporting SUV values should denote which, if any, anthropomorphic measure was used for correction. The uptake measured in a target area can also be compared with another area, leading to uptake parameters such as target-to-background ratio (or SUV ratio) and target-to-blood ratio. Target–background calculations may be different between studies, as it depends on the background chosen, and therefore those studies cannot be combined. For example, in the studies that examined ankylosing spondylitis, some studies used the contralateral SI-joint as background bone for the target-to-background ratio, while others used the body of the L5 vertebra.

Furthermore, the [^18^F]NaF PET protocol used in the different studies showed variation regarding injected dose and the time interval between injection and scan itself. The European Association of Nuclear Medicine (EANM) has established practice guidelines for standardization, which describe recommended image acquisition parameters. The EANM [^18^F]NaF guidelines of 2015 recommend an injected dose of 1.5–3.7 mBq/kg (up to max 370 mBq) in adults and 2.2 mBq/kg (up to max 185 mBq) in children [7]. 11% (10/92) of the studies included appear to have exceeded the maximum injected recommended dose and were published after 2015. The interval between injection and PET scan varied from 30 to 90 min. Tracer uptake is known to depend on time interval and therefore the European Organization for Research and Treatment of Cancer (EORTC) and National Cancer Institute (NCI) provided guidelines in the recommended time interval for the [^18^F]FDG PET response criteria in solid tumors (PERCIST) is 60 min with an acceptable range of 55 to 75 min [77] and 60 ± 10 min [78, 79]. The EANM procedure guidelines for bone imaging, however, do not provide details on this, merely stating that a static scan should be performed at least 30–40 min after tracer injection when the time–activity curve of the bone changes slowly.

We have provided a list of information studies using the [^18^F]NaF PET/CT should consider to include to adequately interpret quantitative study results and make [^18^F]NaF PET studies uptake a reliable and reproducible as a clinical marker for future studies. To also ensure reliability and comparability between different PET systems and across sites, international harmonization programs such as EARL have been developed. A combination of standardization of PET acquisition and analysis and harmonization should minimize the inconsistency in quantitative outcomes across sites and different scanners.

In recent years, long-axial field-of-view (LAFOV) PET/CT scanners have been developed. As the name suggests, LAFOV PET/CT scanners can capture large anatomical areas in a single scan with a field of view exceeding 100 cm, thereby offering several advantages over regular PET scanners [80]. A larger field of view allows for faster acquisition of whole-body PET images with less administered radioactivity, generating images with a higher detection sensitivity and a higher temporal resolution [81]. Moreover, by performing dynamic scans with LAFOV PET/CT scanners, it is possible to obtain tracer kinetics over the whole-body instead over the circa 30 cm available in conventional PET scans. It is likely that in the future, full quantitative kinetic analysis will yield more information than single time-point semi-quantitative analysis and may, therefore, be preferred in a research setting. Studies have also shown that these advanced PET/CT systems can obtain excellent PET images with lower tracer dosages and thereby reducing radiation burden for patients [82]. However, these scanners will not be readily available in a routine clinical practice and from a patient’s perspective, underlining the necessity to continue to perform validation of semi-quantitative markers with full dynamic analysis.

In conclusion, this systematic review illustrates substantial heterogeneity in [^18^F]NaF PET quantification methodology between studies. Ultimately, quantitative parameters derived from [^18^F]NaF PET scans should be carefully examined for sensitivity, specificity, accuracy, validity, and reproducibility.

## Supplementary Information

Below is the link to the electronic supplementary material.

Supplementary file1 (DOCX 17 KB)

Supplementary file2 (XLSX 18 KB)

## Data Availability

Data sharing is not applicable to this article as no datasets were generated or analysed during the current study.
