# Supplementary material for: Validation of quantitative [18F]NaF PET uptake parameters in bone diseases: a systematic review
Source: Ann Nucl Med. 2024 Dec 27;39(2):98–149. doi: 10.1007/s12149-024-01991-9 (PMC11799077; doi:10.1007/s12149-024-01991-9)
Supplement: Supplementary file 1 — Supplementary file1 (DOCX 17 KB) [file 12149_2024_1991_MOESM1_ESM.docx]

# Appendix 1

## PubMed (585)

| **Search** | **Query** | **Results** |
| --- | --- | --- |
| #7 | **#6 NOT ("Animals"[Mesh] NOT "Humans"[Mesh])** | [585](https://pubmed.ncbi.nlm.nih.gov/?term=%236+NOT+%28%22Animals%22%5BMesh%5D+NOT+%22Humans%22%5BMesh%5D%29&sort=relevance&ac=no) |
| #6 | **#5 NOT #4** | [670](https://pubmed.ncbi.nlm.nih.gov/?term=%235+NOT+%234&sort=relevance&ac=no) |
| #5 | **#1 AND #2 AND #3** | [1,205](https://pubmed.ncbi.nlm.nih.gov/?term=%231+AND+%232+AND+%233&sort=relevance&ac=no) |
| #4 | **"Neoplasm Metastasis"[Mesh] OR "Neoplasm Invasiveness"[Mesh] OR metast*[tiab] OR micrometast*[tiab] OR seeding*[tiab] OR circulat*[tiab] OR spread*[tiab]** | [1,520,867](https://pubmed.ncbi.nlm.nih.gov/?term=%22Neoplasm+Metastasis%22%5BMesh%5D+OR+%22Neoplasm+Invasiveness%22%5BMesh%5D+OR+metast%2A%5Btiab%5D+OR+micrometast%2A%5Btiab%5D+OR+seeding%2A%5Btiab%5D+OR+circulat%2A%5Btiab%5D+OR+spread%2A%5Btiab%5D&sort=relevance&ac=no) |
| #3 | **"Bone and Bones"[Mesh] OR bone*[tiab] OR skelet*[tiab]** | [1,512,621](https://pubmed.ncbi.nlm.nih.gov/?term=%22Bone+and+Bones%22%5BMesh%5D+OR+bone%2A%5Btiab%5D+OR+skelet%2A%5Btiab%5D&sort=relevance&ac=no) |
| #2 | **"Positron Emission Tomography Computed Tomography"[Mesh] OR "positron emission*"[tiab] OR PET[tiab]** | [156,599](https://pubmed.ncbi.nlm.nih.gov/?term=%22Positron+Emission+Tomography+Computed+Tomography%22%5BMesh%5D+OR+%22positron+emission%2A%22%5Btiab%5D+OR+PET%5Btiab%5D&sort=relevance&ac=no) |
| #1 | **"Fluorine-18" [Supplementary Concept] OR "naf f18"[tiab] OR "18f fluoride"[tiab] OR "sodium fluoride*"[tiab] OR "18f naf"[tiab] OR "fluorine 18"[tiab] OR "fluor 18"[tiab] OR "fluoride 18"[tiab] OR "fluoride F 18"[tiab] OR "fluorine complex F 18"[tiab] OR "fluorine F 18"[tiab] OR "radiofluoride"[tiab] OR "radiofluorine 18"[tiab]** | [12,729](https://pubmed.ncbi.nlm.nih.gov/?term=%22Fluorine-18%22+%5BSupplementary+Concept%5D+OR+%22naf+f18%22%5Btiab%5D+OR+%2218f+fluoride%22%5Btiab%5D+OR+%22sodium+fluoride%2A%22%5Btiab%5D+OR+%2218f+naf%22%5Btiab%5D+OR+%22fluorine+18%22%5Btiab%5D+OR+%22fluor+18%22%5Btiab%5D+OR+%22fluoride+18%22%5Btiab%5D+OR+%22fluoride+F+18%22%5Btiab%5D+OR+%22fluorine+complex+F+18%22%5Btiab%5D+OR+%22fluorine+F+18%22%5Btiab%5D+OR+%22radiofluoride%22%5Btiab%5D+OR+%22radiofluorine+18%22%5Btiab%5D&sort=relevance&ac=no) |

## Embase (683)

| **Search** | **Query** | **Results** |
| --- | --- | --- |
| #8 | #7 NOT ‘conference abstract’/it | 683 |
| #7 | Search: **#6** NOT ([animals]/lim NOT [humans]/lim) | 1,383 |
| #6 | Search: **#5 NOT #4** | 1,727 |
| #5 | Search: **#1 AND #2 AND #3** | 3,144 |
| #4 | 'metastasis'/exp **OR 'tumor invasion'/exp OR (metast* OR micrometast* OR seeding* OR circulat* OR spread*):ti,ab,kw** | 2,192,547 |
| #3 | 'bone'/exp **OR (bone* OR skelet*):ti,ab,kw** | 2,047,217 |
| #2 | 'positron emission tomography-computed tomography'/exp **OR ("positron emission*" OR PET):ti,ab,kw** | 275,873 |
| #1 | **'fluorine 18'/exp OR ("naf f18" OR "18f fluoride" OR "sodium fluoride*" OR "18f naf" OR "fluorine 18" OR "fluor 18" OR "fluoride 18" OR "fluoride F 18" OR "fluorine complex F 18" OR "fluorine F 18" OR "radiofluoride" OR "radiofluorine 18"):ti,ab,kw** | 30,502 |

## Web of Science (848)

| **Search** | **Query** | **Results** |
| --- | --- | --- |
| #6 | **#5 NOT #4** | 848 |
| #5 | **#1 AND #2 AND #3** | 1,584 |
| #4 | **TS=(metast* OR micrometast* OR seeding* OR circulat* OR spread*)** | 1,972,898 |
| #3 | **TS=(bone* OR skelet*)** | 1,411,175 |
| #2 | **TS=("positron emission*" OR PET)** | 268,914 |
| #1 | **TS=("naf f18" OR "18f fluoride" OR "sodium fluoride*" OR "18f naf" OR "fluorine 18" OR "fluor 18" OR "fluoride 18" OR "fluoride F 18" OR "fluorine complex F 18" OR "fluorine F 18" OR "radiofluoride" OR "radiofluorine 18")** | 15,607 |
